# Ring-Opening Polymerization of *L*-Lactide Catalyzed by Potassium-Based Complexes: Mechanistic Studies

**DOI:** 10.3390/polym14152982

**Published:** 2022-07-23

**Authors:** Christian Rentero, Jesús Damián, Asier Medel, María Fernández-Millán, Yolanda Rusconi, Giovanni Talarico, Tomás Cuenca, Valentina Sessini, Marta E. G. Mosquera

**Affiliations:** 1 Department of Organic and Inorganic Chemistry, Institute of Chemical Research “Andrés M. del Río” (IQAR), Universidad de Alcalá, Campus Universitario, 28871 Madrid, Spain; christian.rentero@edu.uah.es (C.R.); jesus.damian@edu.uah.es (J.D.); asier.medel@edu.uah.es (A.M.); fernandezmillan.maria@gmail.com (M.F.-M.); tomas.cuenca@uah.es (T.C.); 2 Dipartimento di Scienze Chimiche, Università degli Studi di Napoli Federico II, Via Cintia, 80124 Napoli, Italy; yolandarusconi@gmail.com (Y.R.); talarico@unina.it (G.T.)

**Keywords:** catalyst design, earth-abundant metal catalysis, sustainable chemistry, green catalysis

## Abstract

Two non-toxic potassium compounds, **1** and **2**, with a commercial oximate ligand have been prepared and fully spectroscopically characterized. Their activity as catalysts for the ring-opening polymerization (ROP) process of LLA has been studied, showing that they are extremely active and able to polymerize the monomer in a few minutes. For derivative **2**, the presence of a crown ether in the potassium coordination sphere affects the nuclearity of the compound and consequently its solubility, with both aspects having an influence in the polymerization process. Detailed studies of the polymerization mechanism have been performed, and an unusual anionic mechanism was observed in absence of a co-initiator. Indeed, the monomer deprotonation generates a lactide enolate, which initiates the polymerization propagation. On the contrary, when a 1:1 ratio of cat:BnOH is used, a mixture of mechanisms is observed, the anionic mechanism and the activated monomer one, while from a cat:BnOH ratio of 1:2 and over, only the activated monomer mechanism is observed.

## 1. Introduction

In the last few decades, bio-based polymers have attracted a lot of attention as suitable candidates to substitute the fossil-derived counterparts [1]. Within bio-based polymers, PLA stands out due to its multifunctional properties and multiple applications [2]. Although PLA has a lower thermal resistance and poorer processability in comparison to conventional plastics, the fact that it can be obtained from renewable sources together with its biocompatibility and biodegradability makes PLA an attractive option to substitute them [3,4]. Since physical, mechanical and degradation properties of PLA can be tuned by controlling its microstructure, including its molecular orientation and crystallinity [5], the choice of an appropriate polymerization method is essential. In addition, due to its intrinsic chirality, PLA can display advanced properties such as piezoelectricity [6].

One of the most popular methodologies to obtain PLA is through ring-opening polymerization (ROP) of the cyclic ester lactide using metal catalysts. In our research, we are interested in developing active catalysts for the ROP process of lactide to give PLA with a controlled microstructure using non-toxic metals [7,8]. Since PLA can be used for different biomedical applications, the choice of a non-toxic metal catalyst is preferable. Amongst those, potassium is especially interesting since not only is it non-toxic, but it is also an Earth-abundant metal. Although potassium derivatives have been less explored as ROP catalysts than the ubiquities aluminum or zinc compounds, we and others have shown the high activity of some potassium complexes in the ROP of lactide [9,10]. In comparison to other alkali metals such as Li or Na, a clear superior activity for the potassium compounds is observed in all the studies [8].

In most reported cases of potassium complexes as ROP catalysts, the ligands present are aryloxide [11,12,13,14,15,16,17,18,19,20] and less frequently O- and/or N-donors such as amino-phenolates [21,22] amidinates [23] and quinolinolates [24]. Other neutral donor ligand such as sulfonamides [25] or calixarenes [26] have also been explored. In marked contrast, little is known about the possibilities of other interesting N- and O-donor ligands, such as the oximates. This type of ligand is characterized by the presence of two donor atoms next to each other; hence, it can show different coordination modes as well as an interesting reactivity due to cooperative effects [27,28]. In addition, oximate groups are present in quite a few biological processes, and they are used for chemical decontamination applications. Indeed, oximate ions can catalyze the degradation reaction of organophosphorus compounds, thus providing a convenient approach to combat their toxicity [29].

Nevertheless, a limited number of metal complexes with oximate ligands have been reported in the literature using different transition and block f metals [30,31,32,33,34,35,36,37,38,39,40,41,42,43,44,45]. Only two papers describing potassium complexes using oximate ligands have been published. The first one was published in 1994, where Buncel et al. reported the reaction of three potassium oximates, potassium 2,3-butanedione monoximate, acetophenone oximate, and acetone oximate with P-phenylmercaptoethyl p-nitrophenolate [46]. Quite a few years later, in 2020, our group published several potassium oximate compounds, including those containing chiral ligands derived for limonene, which were shown to be very active catalysts for ROP of lactide [7].

In order to focus our studies on the activity of the oximate functional group in this paper, we present a study of a readily accessible compound, the potassium acetophenone oximate. This derivative unites the lack of toxicity of both the metal and the ligand. In fact, this compound has been used in cosmetic barrier creams [47]. In this study, this accessible non-toxic compound is shown to be a very active catalyst for the polymerization of *L*-lactide. In addition, in order to achieve more controlled polymerization through the tuning of the metal coordination sphere, the potassium oximate bearing the 18-crown-6 ether was also prepared. A detailed structural analysis has been performed. We have also evaluated their activity as initiators in polymerization of *L*-lactide, both in the presence or not of benzyl alcohol as co-initiator, and detailed studies have been carried out to make a proposal of the mechanism for the polymerization reactions.

## 2. Materials and Methods

### 2.1. General Methods

All manipulations were conducted under inert atmosphere using standard Schlenk-line techniques (O_2_ < 3 ppm) in conjunction to a MBraunUnilab-MB-20-G glove box (O_2_ < 0.6 ppm). All solvents were rigorously dried prior to use using an MBraun Solvent Purification System. NMR spectra were recorded at 400.13 (^1^H) and 100.62 (^13^C) MHz on a Bruker AV400, at room temperature. Chemical shifts (δ) are given in ppm using C_6_D_6_, CDCl_3_, DMSO-d_6_ as solvents. ^1^H and ^13^C resonances were measured relative to solvent peaks considering tetramethylsilane (TMS) δ = 0 ppm. When necessary, assignment of the resonances signals was performed from HSQC-ed, HMBC and 2D experiments. Elemental analyses were obtained on a Perkin-Elmer Series II 2400 CHNS/O analyzer.

### 2.2. Syntesis of Homometallic Compounds

#### 2.2.1. Potassium (E)-Acetophenone Oximate, (**1**)

A Schlenk flash was charged by 0.50 g (3.51 mmol) of acetophenone oxime, 0.81 g (3.86 mmol) of [K[N(SiMe_3_)_2_] and 20 mL of toluene. This suspension was stirred for 60 min, at room temperature. The resulting white solid was isolated, washed with n-hexane and characterized. Yield: 0.54 g, 3.12 mmol, 86%. ^1^H NMR (298 K, DMSO-d_6_): 1.87 (s, 3H, CH_3_), 6.90 (m, 2H, H_para_), 7.11 (m, 2H, H_meta_), 7.54 (m, 2H, H_orto_). ^13^C {^1^H} NMR (298 K, DMSO-d_6_): 9.9 (s, CH_3_), 121.4 (s, C_orto_), 122.4 (s, C_meta_), 127.3 (s, C_para_), 143.0 (s, C_ipso._), 144.1 (s, C-NOK). E.A. Calc. (%) for C_8_H_8_KNO (173.25 g·mol^−1^): C, 55.46; H, 4.65; N, 8.09. Exp. (%): C, 54.76; H, 4.62; N, 8.11.

#### 2.2.2. Potassium (18-Crown-6 Ether) (E)-Acetophenone Oximate, (**2**)

A Schlenk flash was charged by 0.20 g (1.41 mmol) of acetophenone oxime, 0.33 g (1.55 mmol) of K[N(SiMe_3_)_2_] and 20 mL of toluene. This suspension was stirred for 60 min, at room temperature. After this time, a solution of 1 equiv. (0.37 g) of 18-crown-6 ether in 10 mL of toluene was added into the reaction flask. Finally, the resulting mixture was filtered, isolating a yellow solid, which was purified with n-hexane and characterized. Yield: 0.59 g, 1.34 mmol, 90%. ^1^H NMR (298 K, DMSO-d_6_): 1.87 (s, 3H, CH_3_), 3.54 (s, 24H, 18-crown-6 ether), 6.90 (m, 1H, H_para_), 7.10 (m, 2H, H_meta_), 7.55 (m, 2H, H_orto_). ^13^C {^1^H} NMR (298 K, C_6_D_6_): 11.2 (s, CH_3_), 70.3 (s, 18-crown-6 ether), 123.6-123.7 (s, C_orto_+C_meta_), 128.0 (s, C_para_), 144.3 (s, C_ipso._), 146.7 (s, C-NOK). E.A. Calc. (%) for C_20_H_32_KNO_7_ (437.56 g·mol^−1^): C, 54.90; H, 7.37; N, 3.20. Exp. (%): C, 53.38; H, 6.92; N, 3.29.

### 2.3. Single-Crystal X-ray Structure Determination

Data collection was performed at 200(2) K, with the crystals covered with perfluorinated ether oil. Single crystals of **2** and **2·H_2_O** were mounted on a Bruker D8 Venture single crystal diffractometer equipped with a Mo-*K*_α_ radiation (*λ* = 0.71073 Å). Multiscan [48] absorption correction procedures were applied to the data. The structure was solved using the WINGX package [49], by direct methods (SHELXS-13), and refined using full-matrix least-squares method against *F*^2^(SHELXL-16) [50,51]. Compound **2** crystallizes in a non-centrosymmetric space group with a Flack parameter of 0.01(9), two identical molecules are present in the asymmetric unit. For both compounds, all non-hydrogen atoms were anisotropically refined. Hydrogen atoms were geometrically placed and left riding on their parent atoms. Full-matrix least-squares refinements were carried out by minimizing Σw(*Fo*^2^ − *Fc*^2^)^2^ with the SHELXL-97 weighting scheme and stopped at shift/err < 0.001. The final residual electron density maps showed no remarkable features. (See SI for the crystallographic tables.)

### 2.4. Polymerization Procedure

The preparation of solutions and all the polymerization experiments were carried out under inert atmosphere in the glovebox or in the vacuum line, while the treatment and purification of the polymers have been performed in air. In all cases, the solvent used was toluene.

The purification of the monomer was carried out by recrystallization in toluene and sublimation, at 100 °C, and then, the purified lactide was stored in the glovebox. The manipulation of other reagents was also effectuated in the glovebox.

For polymerization experiments, two different strategies were developed. On one hand, the method in presence of benzyl alcohol (BnOH) consisted of the addition of a solution that contained the catalyst and the co-initiator to a *L*-lactide suspension. When the reaction mixture was completely dissolved, an aliquot was removed with a syringe to determine the conversion by ^1^H NMR in CDCl_3_. On the other hand, in absence of BnOH, the catalyst in solid state was added to the monomer solution. In this instance, an aliquot was removed with a syringe at different times to determine the conversion by ^1^H NMR in CDCl_3_.

In both methods, when the conversion reached the value sought for, normally 100%, the polymerization was stopped by adding hexane, which caused the precipitation of the polymer. Finally, the product was purified in a mixture CH_2_Cl_2_/n-hexane and the polymer was filtered and dried under vacuum to constant weight.

### 2.5. Gel Permeation Chromatography (GPC)

The molecular weights (M_n_ and M_w_) and polydispersity (Ð = M_w_/M_n_) of the polymers were analyzed by size-exclusion chromatography on an Agilent apparatus. Sample solutions were injected with a 1 mL·min^−1^ flow rate at 30 °C, in a pre-column PL gel 5 µm and in two gradient columns PL gel 5 µm. The mobile phase was tetrahydrofuran (THF) and the equipment was calibrated with respect polystyrene (PS) standards.

### 2.6. Mass Spectroscopy (MS)

The chain end-groups of PLLA were determined by MALDI-TOF experiments. The mass spectra were obtained on Bruker ULTRAFLEX III TOF/TOF, with an NdY AG laser using DCTB as matrix and KI as cationization agent. 

### 2.7. Density Functional Theory (DFT) Calculations

All geometry optimizations were performed using the Gaussian09 set of programs [52], using the B3LYP functional [53,54]. The electronic configuration has been described using a Def2-QZVPP [55] basis set for K and SVP [56] for H, C, N, O, F. Stationary points were characterized using vibrational analysis, and this analysis has been also used to calculate zero-point energies and thermal (enthalpy and entropy) corrections (298.15 K, 1 bar), without further translational corrections [57]. Improved electronic energies were obtained from single-point energy calculations with wB97XD [58] functional and using Def2-QZVPP basis set for K and 6-311G+(d,p) basis set on all the atoms. Solvation effect has been considered in the single-point energy calculations using the SMD model [59]. These energies added to the thermal corrections are named ΔG; this computational protocol has been proved to reach a good agreement with the experimental results on lactide polymerization [60,61]. The counterpoise corrections [62] have been calculated with the same basis sets used for the geometry optimization, specifying the number of fragments composing the structure of interest. 

## 3. Results and Discussion

### 3.1. Synthesis and Behavior of the Metallic Complexes 

**Scheme 1:**
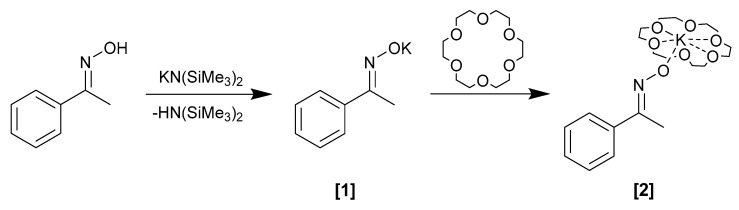
Synthesis of potassium compounds **1** and **2**.

In this work, the potassium acetophenone oximate **1** was synthesized by the treatment of the commercial ligand acetophenone oxime with the potassium precursor KN(SiMe_3_)_2_. In order to prepare the derivative **2** with the 18-crown-6 ether, the reaction was performed similarly, and the crown ether was added *in situ* to the reaction media (Scheme 1).

The formation of the derivatives was confirmed by ^1^H and ^13^C NMR spectra. For compound **1**, the signal corresponding to the oxime proton had disappeared, and the peaks for the methyl group and the aromatic ring had shifted to higher field (see Figures S1 and S2). For compound **2**, a singlet both in the proton and the carbon NMR spectra corresponding to the 18-crown-6 ether are clearly observed at 3.55 ppm and 70.3 ppm, respectively (see Figures S3–S5).

**Figure 1:**
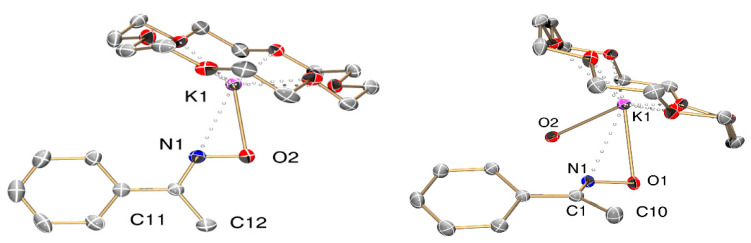
ORTEP view (30% probability ellipsoids) of **2** (**left**) and **2·H_2_O** (**right**). Hydrogen atoms are omitted for clarity.

From compound **2**, appropriate crystals could be isolated to determine the structure in solid state by single-crystal X-ray diffraction. In the asymmetric unit, two identical molecules are present. As shown in Figure 1 left, the oximate acts as bidentate ligand and coordinates to the potassium atom through the oxygen and the nitrogen. The metal–ligand distances are within the range of the observed ones in analogous compounds: K(1)-O(1): 2.637(9) Å, K(1)-N(1): 2.817(9) Å, N(1)-O(1)-K(1): 83.6(3)° for one molecule and K(2)-O(2): 2.609(5), K(2)-N(2): 2.799(6) and N(2)-O(2)-K(2): 83.8(3)° for the other [63]. In the structure, the coordination of the 18-crown-6 ether by its oxygen atoms is also evident, the K-O bond distances are longer than the ones with the oximate and ranges from 2.834(5) to 3.060(5) Å in one molecule and from 2.818(5) to 3.093(5) Å in the other. Interestingly a similar compound bearing the dicyclohexane-18-crown-6-ether had been described by Buncel et al. as an ion pair, although they did not perform a structural determination in the solid state. As shown in the Figure 1, this compound cannot be considered so, since the potassium cation and the oximate are within bonding distances. This may explain the result observed by Buncel, wherein the reactivity of the crown ether compound was clearly different than the one with 2.2.2 cryptand where the ions are truly separated [46].

We also checked the effect of the presence of another O-donor ligand in the coordination sphere and the water solvate **2·H_2_O** was isolated (Figure 1 right). In this case, one water molecule bonds and has some effect in the geometry of the molecule. The K-O bond distance is longer, 2.784(2) Å, than in the absence of the coordinated water molecule, while the K-N distance does not significantly change, 2.8183(19) Å. The K-O distance to the water ligand, 2.8183(19) Å, is within the range for a K-O donor bond. Interestingly, the crown ether has tilted and now the K-O distances range from 2.7600(19) Å to 3.033(2) Å, this can be attributed to the need to accommodate a water molecule. A similar behavior could be expected when a monomer molecule interacts with this compound.

**Figure 2:**
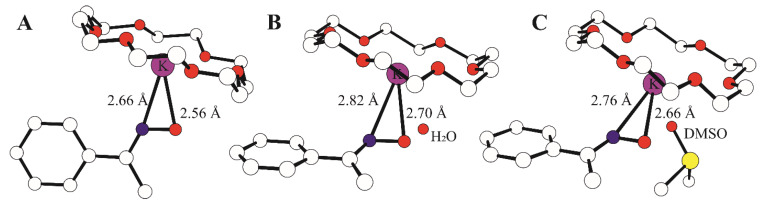
DFT geometries of system **2** optimized in in gas-phase (**A**) and in presence of explicit molecule of solvent (H_2_O, (**B**) and DMSO, (**C**)). Hydrogen atoms omitted for clarity and distances in Å.

Finally, we optimized the structure of **2** by DFT calculations both in gas-phase and in solution. As shown in the Figure 2, the optimized structure was analogous to the resulting one by single-crystal XRD, indicating that this disposition is also the most stable in solution. 

**Figure 3:**
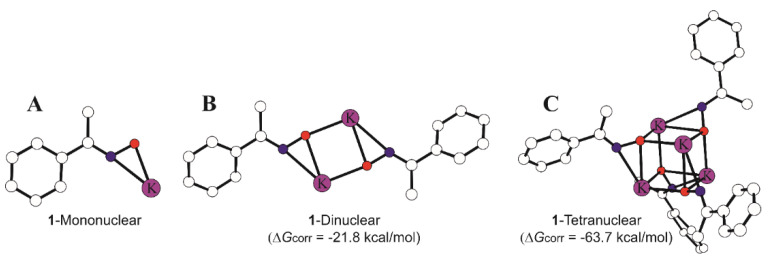
DFT geometries of system **1** optimized in gas-phase for mononuclear (**A**), dinuclear (**B**) and tetranuclear (**C**). The calculated Gibbs energies are corrected by BSSE (Δ*G*_corr_), and the values are referred to as 2 (**1**-Mononuclear) for (**B**) and 4 (**1**-Mononuclear) for (**C**) isolated species. Hydrogen atoms are omitted for clarity.

Since we could not obtain crystals of enough quality to determine the structure of **1** in the solid state, we performed a computational study to check its most stable structure. For **1** without any solvent molecules coordinated, the stability in solution of the mononuclear, dinuclear and tetranuclear species were compared (Figure 3). Surprisingly, it was found that the tetranuclear specie showed extremely high stability compared to four mononuclear and also two dinuclear ones (see Δ*G*_corr_ values in Figure 3). Hence, the most stable structure for **1** may be a cubic core with alternated potassium and oxygen atoms as vertexes having each nitrogen coordinated to one metal center. So, it could be expected this would be the structure present in solution, in the absence of a donor solvent. The picture is clearly different when considering the more crowded system **2**, where we found a (small) preference for the mononuclear species with respect to the dinuclear one (Δ*G*_corr_ = 1.8 kcal/mol, see Figure S14).

### 3.2. L-Lactide Polymerization

We studied the activity of compounds **1** and **2** in different conditions and in the presence or not of a co-initiator such as the benzyl alcohol (BnOH). All the polymerizations were performed at room temperature and using toluene as solvent. Both potassium compounds proved to be very active catalysts for the ROP and were able to polymerize *L*-lactide (LLA) in few minutes. We focused on the polymerization of *L*-lactide as we are interested in preparing isotactic PLLA.

In Table 1, entries 17–20, the results of the experiments in absence of co-initiator are displayed. Although high dispersity values were observed with both catalysts, a better control of the ROP process was achieved with compound **2**, which could be attributed to the presence of the crown ether ligand that modulates the potassium coordination sphere. In all cases, fully isotactic PLLA was obtained as determined by NMR (see Figure S6). 

A direct link between cat:LLA ratio and the resulting molecular weights was observed. Indeed, the increase in the monomer ratio gives higher Mn values, in accordance with a living polymerization. In all cases, the experimental molecular mass is higher than the theoretical one, which agrees with the presence of aggregation processes that prevent all the potassium active centers being accessible, as has been previously observed [7]. This effect is more evident in compound **1**, as expected, since the crown ether contributes to limit the aggregation and in consequence influences the solubility. 

We then studied the polymerization in the presence of BnOH as co-initiator, as shown in Table 1. We explored two different ratios, cat:BnOH 1:2 and 1:5, as a result of the mechanistic studies performed (vide infra). The solubility of both catalysts was significantly increased in the presence of BnOH, and a better control of the polymerization was achieved; in addition, the process could be performed with less amount of solvent. The molecular weights are closer to the expected ones, which would be in agreement with the fact that the benzyl alcohol contributes to reducing the aggregation of the potassium oximates. When using a cat:BnOH ratio of 1:2, the molecular weights are half the expected ones, which may indicate the presence of two growing polymer chains per catalysts. The concentration of the solution seems to be a key factor for the polymerization control, as for the more concentrated solutions, the control of the polymerization is significantly worse, which can be attributed to mass transfer limitations in the reaction media. Because these polymerization processes are very fast, aspects such as mass transfer limitation and solubility proved to be key in attaining a good control over the molecular weights (M_n_ and M_w_) and dispersities (Ð). In the studies performed, a better control is achieved when the process is slower.

With the goal of obtaining higher molecular weight polymers, we carried out the polymerization with a 1:2 cat:BnOH ratio with a higher ratio of monomer. We were able to scale to 1:400:2 and 1:1000:2. For the latter, the polymerization process was significantly slower, but a better control was achieved, and polymers with Mn > 20 kDa with moderate dispersities were attained.

### 3.3. Mechanistic Studies

**Scheme 2:**
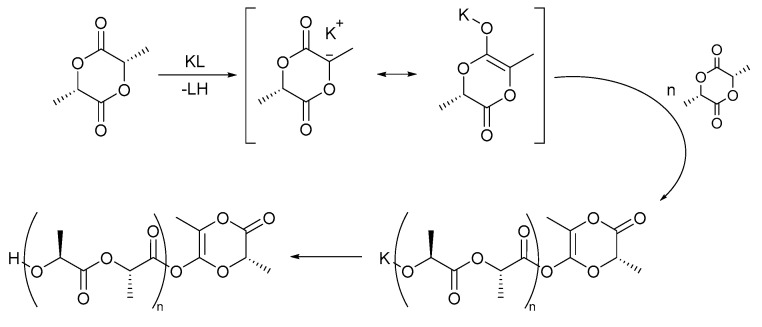
Anionic polymerization mechanism for KL compounds.

**Figure 4:**
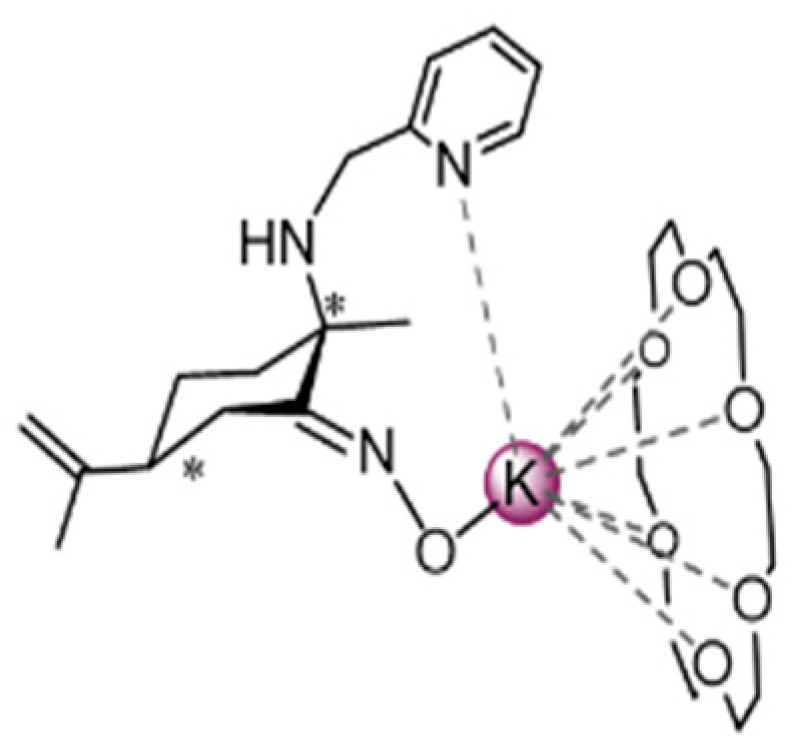
Compound (1*S*,4*R*)-[K(18-crown-6)(L)].

The species described in this paper could be acting via an anionic mechanism, since it is frequently observed for alkali metal initiators (Scheme 2) [64,65,66]. In such a case, a nucleophilic attack of the oximate group to the lactide carbonyl carbon may take place, and the anionic fragment generated would be responsible for the process propagation. In order to check the mechanism, several NMR experiments in different ratios of co-initiator (BnOH) were performed. These studies have been performed for both compounds **1** and **2** and also for the potassium oximate compound (1*S*,4*R*)-[K(18-crown-6)(L)] previously published by us (Figure 4). For the three compounds, the behavior observed is the same, so we will describe in detail only the results obtained for (1*S*,4*R*)-[K(18-crown-6)(L)] and **2** with initiator:monomer:BnOH ratios of 1:1:0, 1:1:1, 1:1:2 and 1:1:5.

#### 3.3.1. Initiator:monomer:BnOH Ratio 1:1:0

**Figure 5:**
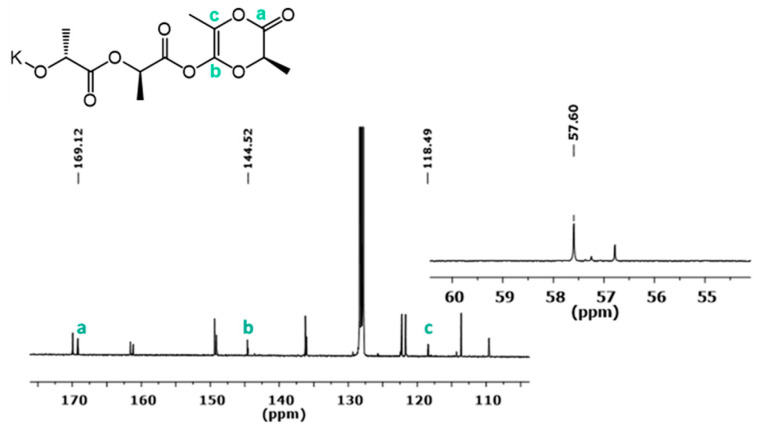
^13^C NMR spectrum for the initiator:monomer:BnOH ratio of 1:1:0 in C_6_D_6_, for catalysts (1*S*,4*R*)-[K(18-crown-6)(L)].

We performed an NMR study in C_6_D_6_, using *L*-lactide as substrate and using (1*S*,4*R*)-[K(18-crown-6)(L)] as the catalyst. In the ^1^H NMR spectrum, the presence of the protonated ligand HL is observed (see Figure S7). This observation shows that the lactide has been deprotonated by the oximate ligand, and hence, an enolate from the lactide has been generated. The formation of such species was confirmed by ^13^C NMR (Figure 5) spectrum, where a signal at 57.6 ppm corresponding to the methyl group of one of the resonant forms of the lactide enolate is present [65,67,68]. Furthermore, other bands at 118.5, 144.5 and 169.1 ppm are observed, corresponding to quaternary carbon centers of the same species and confirming the formation of the enolate. A similar result was obtained when compound **2** is used as catalysts (see Figure S8).

**Figure 6:**
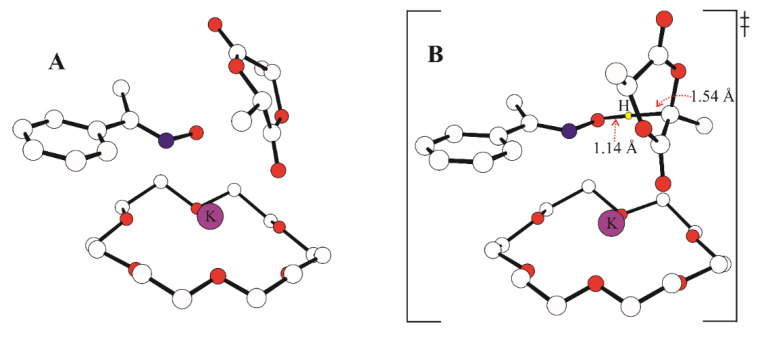
DFT optimized structures for the coordination (**A**) and the monomer deprotonation TS by the oximate group (**B**) calculated for system **2**. The difference in Gibbs energy is 3.6 kcal/mol. Hydrogen atoms omitted for clarity except for the one transferred from the lactide monomer (in yellow). Distances in Å.

This result agrees with an anionic mechanism for the polymerization via the monomer deprotonation, then the resonant form of the enolate initiates the polymerization propagation (Scheme 2). This result show that the basic character of the oximate group is prevalent to the nucleophilic one and the deprotonation takes places instead of the nucleophilic attack to the carbonyl group of the monomer. Since the same behavior has been observed in all cases, being different ligands, this behavior may be ascribed to the highly basic character of the ligands motivated by a strong ionic component of the bond with the potassium. A further confirmation was suggested by DFT calculations of the transition state (TS) structure for the monomer deprotonation (see Figure 6), whose Gibbs energies with respect to the lactide π-complex are very low (3.6 kcal/mol).

Since for alkali metals there is a tendency to form cyclic polymers due to intramolecular transesterification processes, particularly when a co-initiation is not used, we checked if, in this case, these processes had taken place. Usually, MALDI-TOF mass spectroscopy is an appropriate method to determine if a cyclic polymer has been formed (see Figure S17). However, as the terminal groups for these polymers would be a monomer in the enolate form, this technique is not appropriate. Fortunately, in the ^1^H NMR spectrum for the polymer obtained with conditions initiator:BnOH ratio of 1:0, it was possible to identify the hydrogen atom from the CH of the chain end-group -CH(CH_3_)OH (see Figures S9 and S10). Hence, we could confirm that the polymer obtained has a linear structure with enolate and -OH end-groups in agreement with the mechanism proposed in Scheme 2.

#### 3.3.2. Initiator:Monomer:BnOH Ratio 1:1:1

To check the influence of the co-initiator we performed the reaction with one equivalent of BnOH. In the spectrum, the presence of the OH group for the BnOH is evident; hence, there is not a transfer of a proton from the BnOH to the oximate group, in agreement with the stronger acidic character of the oxime proton (pKa = 11.1) in comparison to the benzyl alcohol (pKa =15.0). Furthermore, a diffusion experiment DOSY-2D was performed, where it is clearly observed that both compounds diffuse at different rates (see Figure S13). 

**Scheme 3:**
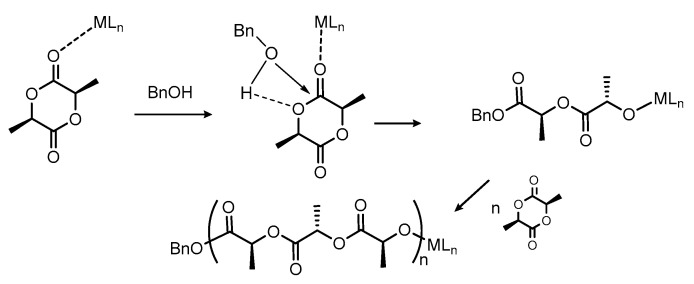
Activated monomer mechanism for lactide polymerization.

To the initiator/co-initiator mixture, one equivalent of monomer was added. In the ^13^C NMR spectrum of the reaction, the signals corresponding to the benzyl group and the enolate groups are present (see Figure S11). Both groups are end-caps of different types of polymer chains. Therefore, when the co-initiator is present in a 1:1 ratio, there is a competitive process in the polymerization initiation and two different mechanisms are happening simultaneously: the anionic one, which leads to an enolate terminal group (Scheme 2), and an activated monomer mechanism that generates benzyl terminal groups (Scheme 3). 

#### 3.3.3. Initiator:Monomer:BnOH Ratio 1:1:5 and 1:1:2

**Figure 7:**
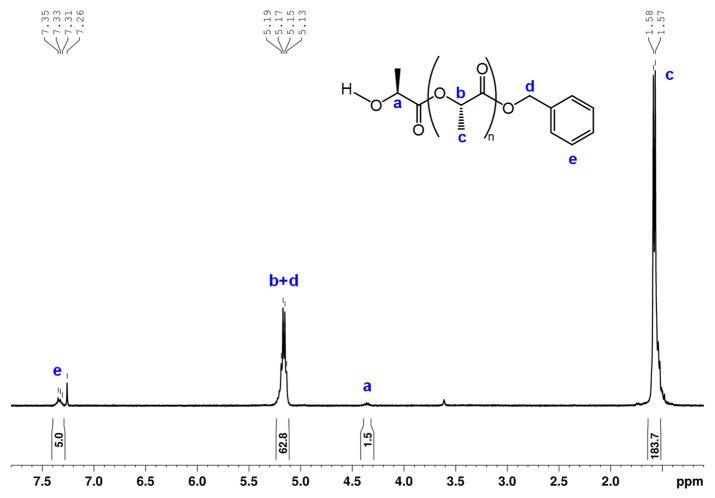
^1^H NMR spectrum for the **2**:monomer:BnOH ratio of 1:100:5 in CDCl_3_.

Considering these observations, we performed the experiment with five equivalents of the co-initiator. In the ^13^C NMR, only the bands corresponding to benzyl end groups are observed (see Figure S12). In addition, by ^1^H NMR, the chain end-caps can be identified as a benzyl and a OH group. The band corresponding to the proton of the CH(a) group bonded to the hydroxyl groups of the end group CH(CH_3_)OH appears as a quadruplet at 4.37 ppm, as shown in Figure 7 (the spectrum corresponds to a 1:100:5 ratio that presents more defined signals). At low field, the resonances corresponding to the five hydrogen atoms of the aromatic ring from the benzyl fragment appear. Hence, the polymers formed when there is an excess of co-initiator are lineal in agreement with an activated monomer mechanism. The MALDI-TOF experiments also corroborates the presence of a benzyloxide moiety as an end-cap group (see Figure S18).

**Figure 8:**
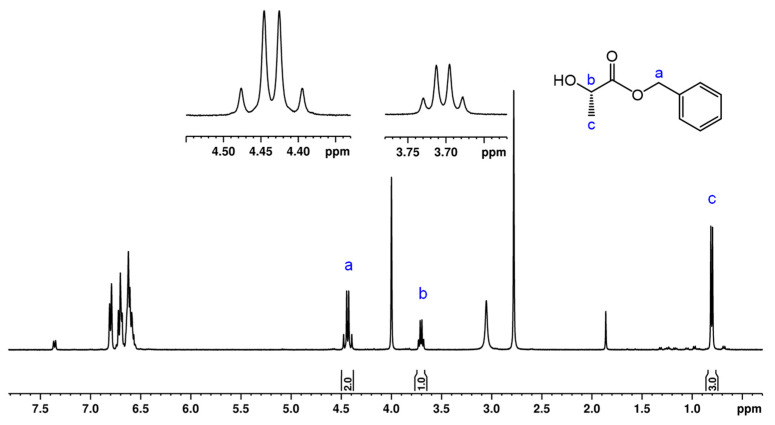
^1^H NMR spectrum for the 1:1:2 ratio for **2**:LLA:BnOH in C_6_D_6_.

In order to explore when the activated monomer mechanism takes over, we reduced the BnOH ratio to 2; in this case, only the activated monomer mechanism is active, as shown in the ^1^H NMR (Figure 8), where the signals for the benzyl lactate are evident. The MS analysis also corroborates this mechanism since the main sequence observed, [(C_3_H_4_O_2_)_n_ + (C_7_H_8_O)] + K^+^, is related to series of lactic acid repetitions with a hydrogen and the benzyloxide as end-groups (see Figure S19).

## 4. Conclusions

Readily available non-toxic potassium oximate compounds have been prepared using acetophenone oximate as ligand and a crown ether. These derivatives are extremely active catalysts for the LLA polymerization via a ROP process and are able to polymerize LLA within minutes. The presence of a crown ether allows a more controlled environment of the metal, as well as influences the nuclearity of the compound and, in consequence, the solubility. Both aspects have a clear effect in the polymerization process, improving its control in absence of the co-initiator, and also influencing the attained molecular weights.

For the potassium oximates described, an unusual polymerization mechanism was observed when no co-initiator is used, via the monomer deprotonation, to generate a lactide enolate, as determined by NMR experiments. This would imply that the polymerization happens through an anionic mechanism. When using a 1:1 ratio, a mixture of mechanisms is observed, and both the anionic and the activated monomer mechanisms are present. From a cat:BnOH ratio of 1:2 and over, only the activated monomer mechanism is detected.

## Figures and Tables

**Table 1:** Results of LLA polymerization ^1^.

Ent.	Cat.	[Cat]:[LLA]: [BnOH]	[LLA] (M)	Conv. (%) ^2^	t (min) ^2^	M_n_ theo. (kDa) ^5^	M_n_ exp. (kDa) ^6^	Ð ^6^
1	[1]	1:100:5	0.2	>99	1	2.99	3.83	1.95
2	[2]	1:100:5	0.2	>99	1	2.99	1.60	1.94
3	[1]	1:100:5	0.8	>99	1	2.99	3.28	1.89
4	[2]	1:100:5	0.8	>99	1	2.99	2.40	4.06
5	[1]	1:200:5	0.2	>99	1	5.87	6.32	1.77
6	[2]	1:200:5	0.2	>99	1	5.87	4.79	1.63
7	[1]	1:200:5	0.8	>99	1	5.87	9.48	3.10
8	[2]	1:200:5	0.8	>99	1	5.87	2.82	2.64
9	[1]	1:100:2	0.8	>99	1	7.32	5.94	2.16
10	[2]	1:100:2	0.8	>99	1	7.32	5.63	1.99
11	[1]	1:200:2	0.8	>99	1	14.52	7.24	2.18
12	[2]	1:200:2	0.8	>99	1	14.52	7.72	2.69
13	[1]	1:400:2	0.8	>99	1	28.93	9.68	3.29
14	[2]	1:400:2	0.8	>99	1	28.93	13.24	3.08
15	[1]	1:1000:2	0.8	35 ^3^	1 ^3^	29.66	29.25	1.35
16	[2]	1:1000:2	0.8	33 ^4^	1 ^4^	25.33	21.54	1.78
17	[1]	1:100:0	0.4	>99	1	14.41	55.83	2.98
18	[2]	1:100:0	0.4	>99	1	14.41	20.35	1.76
19	[1]	1:200:0	0.4	>99	8	28.83	113.66	2.67
20	[2]	1:200:0	0.4	>99	2	28.83	43.53	2.14

^1^ Solvent: toluene. ^2^ Conversion values at different times were calculated by ^1^H NMR in CDCl_3_. There was ^3^ 41% conversion at 16 min and ^4^ 35% conversion at 8 min. ^5^ Mn theorical was calculated considering conversion and benzyl alkoxide as chain end-group. ^6^ Values obtained by GPC (see Figures S15 and S16).

## Data Availability

The data presented in this study are available on request from the corresponding authors.

## Supplementary Materials

The following supporting information can be downloaded at: https://www.mdpi.com/article/10.3390/polym14152982/s1, Figure S1. ^1^H NMR (298K, DMSO-*d_6_*): (E)-acetophenone oxime; Figure S2. ^1^H NMR (298K, DMSO-*d_6_*): Potassium (E)-acetophenone oximate (**1**); Figure S3. ^13^C {^1^H} NMR (298K, DMSO-*d_6_*): Potassium (E)-acetophenone oximate, (**1**); Figure S4. ^1^H NMR (298K, DMSO-*d_6_*): Potassium (18-crown-6 ether) (E)-acetophenone oximate, (**2**); Figure S5. ^13^C {^1^H} NMR (298K, C_6_D_6_): Potassium (18-crown-6 ether) (E)-acetophenone oximate, (**2**); Figure S6. ^1^H NMR (298K, CDCl_3_): Spectrum for washed polymer of enter 20 (**2**:LLA:BnOH ratio 1:200:0); Figure S7. ^1^H NMR (298K, CDCl_3_): Study of **2**:LLA:BnOH ratio 1:1:0; Figure S8. ^13^C {^1^H} NMR (298K, C_6_D_6_): Study of **2**:LLA:BnOH ratio 1:1:0; Figure S9. ^1^H NMR (298 K, C_6_D_6_): Study of (1S,4R)-[K(18-crown-6)(L)]:LLA:BnOH ratio 1:100:0 at −70 °C, before reaching full conversion; Figure S10. ^1^H NMR (298K, CDCl_3_): Spectrum ampliation for washed polymer of enter 20 (**2**:LLA:BnOH ratio 1:200:0); Figure S11. ^13^C NMR spectrum for the (1*S*,4*R*)-[K(18-crown-6)(L)]:LLA:BnOH ratio 1:1:1 in C_6_D_6_; Figure S12. ^13^C NMR spectrum for the (1*S*,4*R*)-[K(18-crown-6)(L)]:LLA:BnOH ratio 1:1:5 in C_6_D_6_; Table S1: Crystal data and structure refinement for 2 and 2·H_2_O; Table S2: Bond Lengths (Å) and Angles (°) for 2; Table S3: Bond Lengths (Å) and Angles (°) for 2·H_2_O; Figure S13. 2D 1H DOSY NMR experiment for the (1S,4R)-[K(18-crown-6)(L)]/BnOH mixture; Figure S14. DFT geometries of system 1 optimized in gas-phase for mononuclear (A) and dinuclear (B) species. The calculated Gibbs energies are corrected by BSSE (Δ*G*_corr_ = 1.8 kcal/mol) showing a preference for mononuclear specie (A). Hydrogen atoms omitted for clarity; Figure S15. Chromatogram example for polymer of enter 6 (**2**:LLA:BnOH ratio 1:200:5); Figure S16. Chromatogram example for polymer of enter 20 (**2**:LLA:BnOH ratio 1:200:0); Figure S17. MALDI-TOF spectrum for polymer of enter 20 (**2**:LLA:BnOH ratio 1:200:0); Figure S18. MALDI-TOF spectrum for polymer of enter 6 (**2**:LLA:BnOH ratio 1:200:5); Figure S19. MALDI-TOF spectrum for polymer of enter 12 (**2**:LLA:BnOH ratio 1:200:2).
