# Supplementary material for: Ring-Opening Polymerization of L-Lactide Catalyzed by Potassium-Based Complexes: Mechanistic Studies
Source: Polymers (Basel). 2022 Jul 23;14(15):2982. doi: 10.3390/polym14152982 (PMC9329769; doi:10.3390/polym14152982)
Supplement: Supplementary file 1 [file polymers-14-02982-s001.zip › polymers-1811884-supplementary.pdf]

Supplementary Material

Ring-Opening Polymerization of *L*-lactide catalyzed by potassium-based complexes: mechanistic studies

Christian Rentero[1], Jesús Damián Burgoa[1], Asier Medel[1], Maria Fernandez Millan[1], Yolanda Rusconi[2], Giovanni Talarico[2], Tomás Cuenca[1], Valentina Sessini[1,*] and M.E.G. Mosquera[1*]

[1] Department of Organic and Inorganic Chemistry, Institute of Chemical Research "Andrés M. del Río" (IQAR), Universidad de Alcalá, Campus Universitario, 28871- Alcalá de Henares, Madrid, Spain.
[2] Dipartimento di Scienze Chimiche, Università degli Studi di Napoli Federico II, Via Cintia, 80124, Napoli, Italy
* Correspondence: valentina.sessini@uah.es, martaeg.mosquera@uah.es

Table of content

1. **NMR Spectra**
2. **Diffusion-Ordered NMR Spectroscopy (DOSY)**
3. **Single-Crystal X-Ray Structure Determination**
4. **Density Functional Theory (DFT) calculations**
5. **Polymers characterization**
    - *5.1. Gel Permeation Chromatography (GPC)*
    - *5.2. Mass Spectroscopy (MS)*

1. NMR Spectra

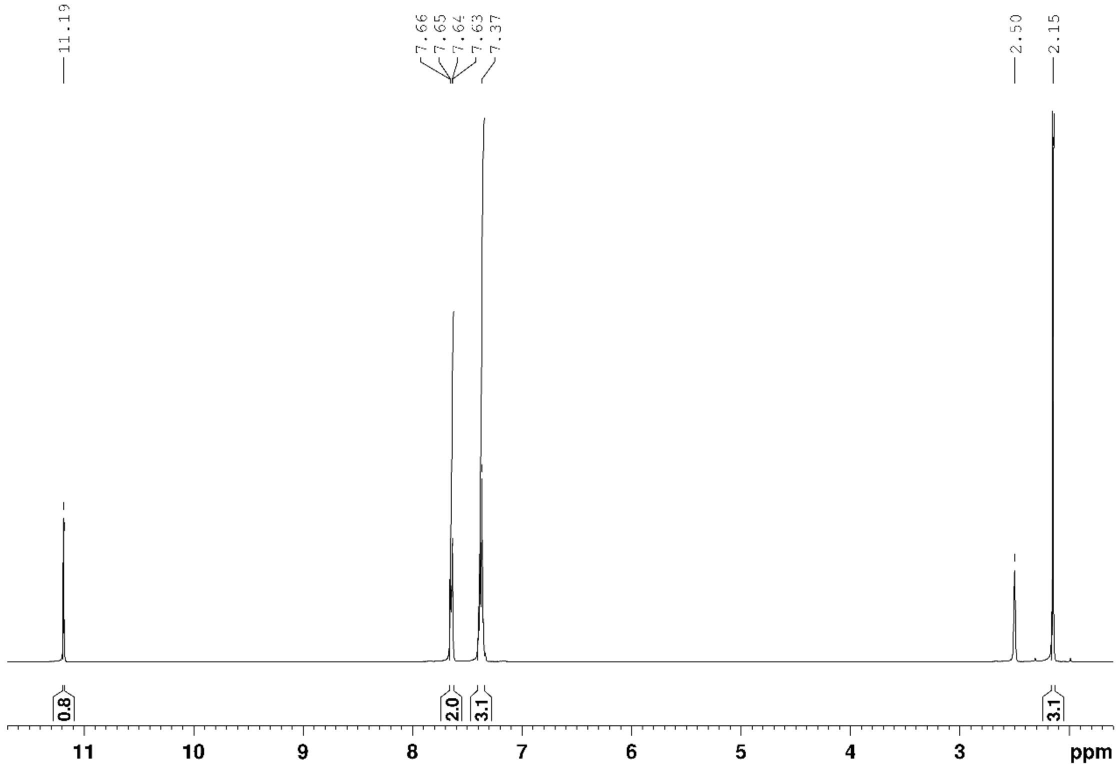

Figure S1. ^{1}H NMR (298K, DMSO-d_6): (E)-acetophenone oxime.

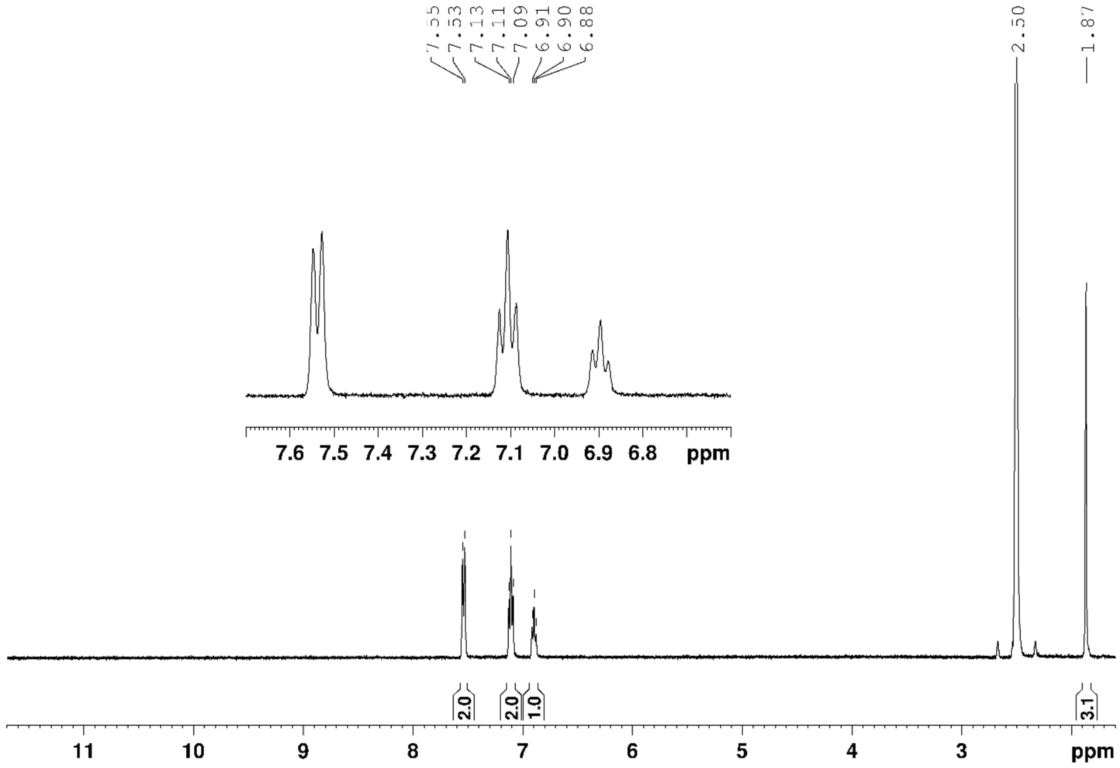

Figure S2. ^{1}H NMR (298K, DMSO-d_6): Potassium (E)-acetophenone oximate (**1**).

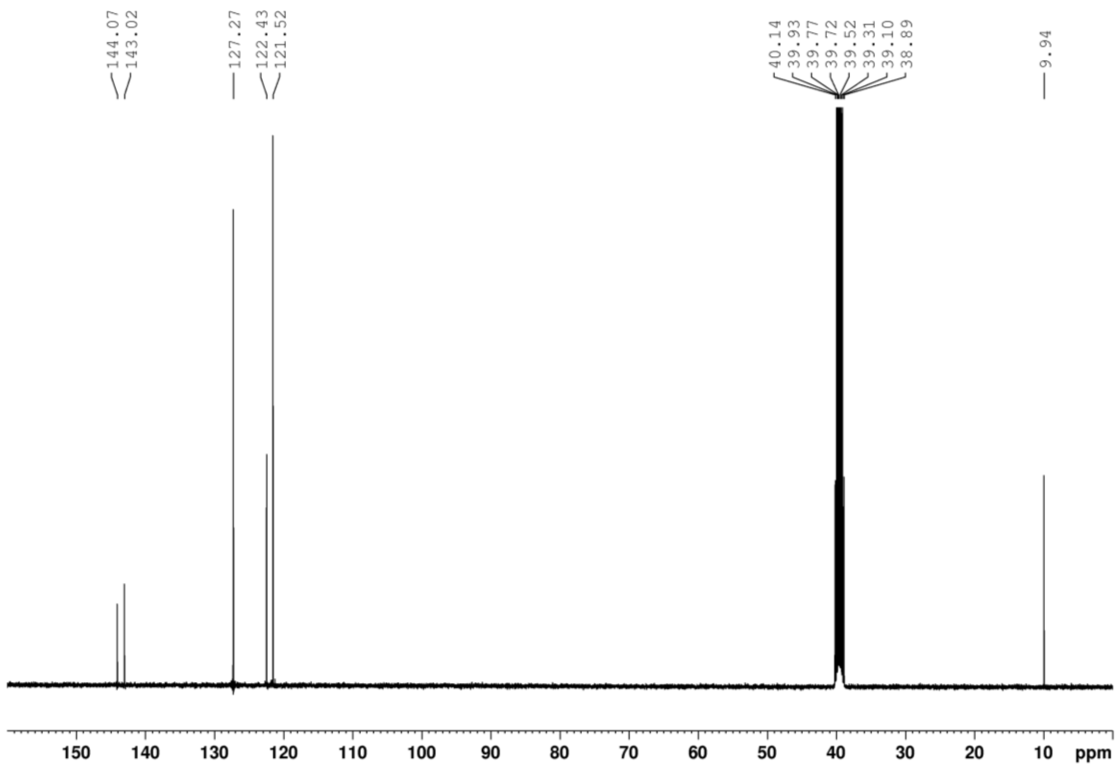

Figure S3. $^{13}C\ \{^{1}H\}$ NMR (298K, DMSO-d_6): Potassium (E)-acetophenone oximate, (**1**).

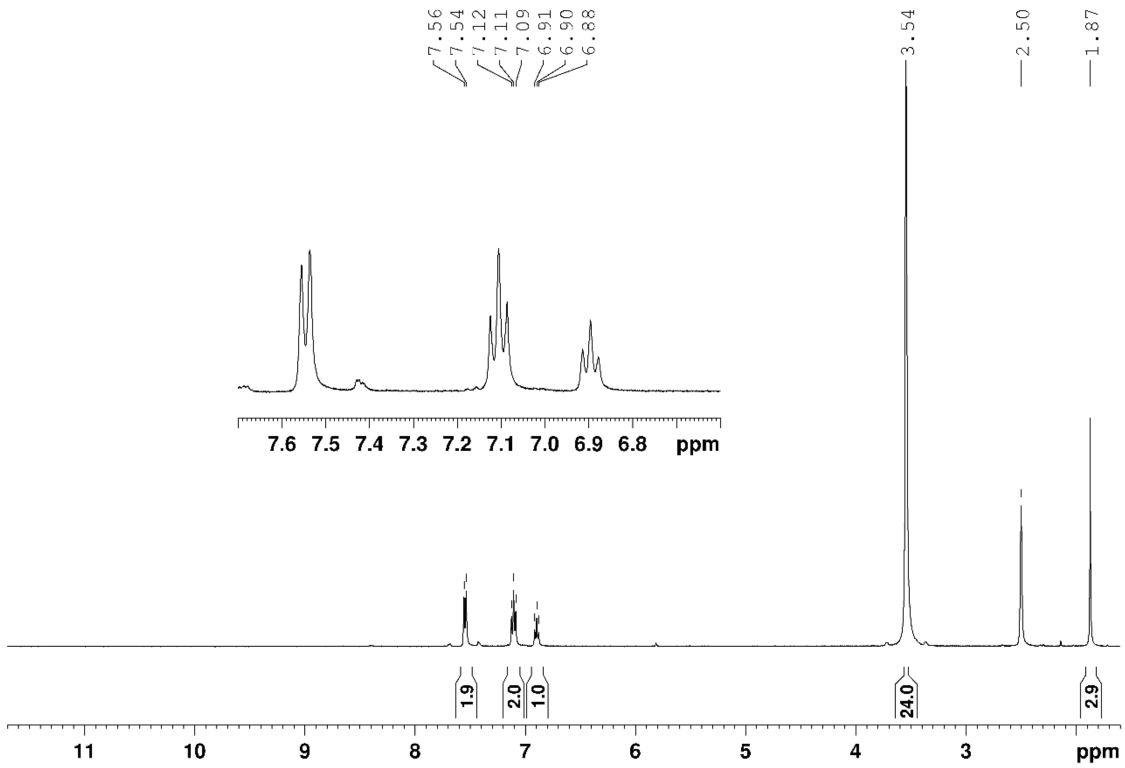

Figure S4. ^{1}H NMR (298K, DMSO-d_6): Potassium (18-crown-6 ether) (E)-acetophenone oximate, (**2**).

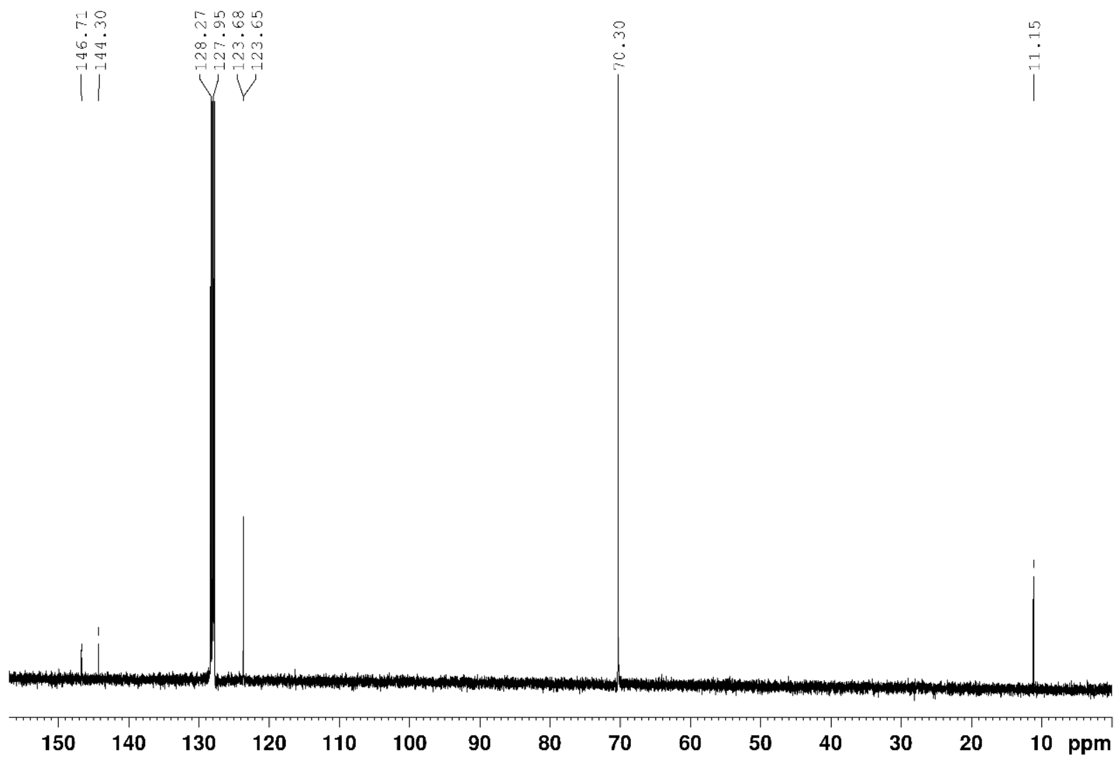

Figure S5. ^{13}C {^{1}H} NMR (298K, C_6D_6): Potassium (18-crown-6 ether) (E)-acetophenone oximate, (**2**).

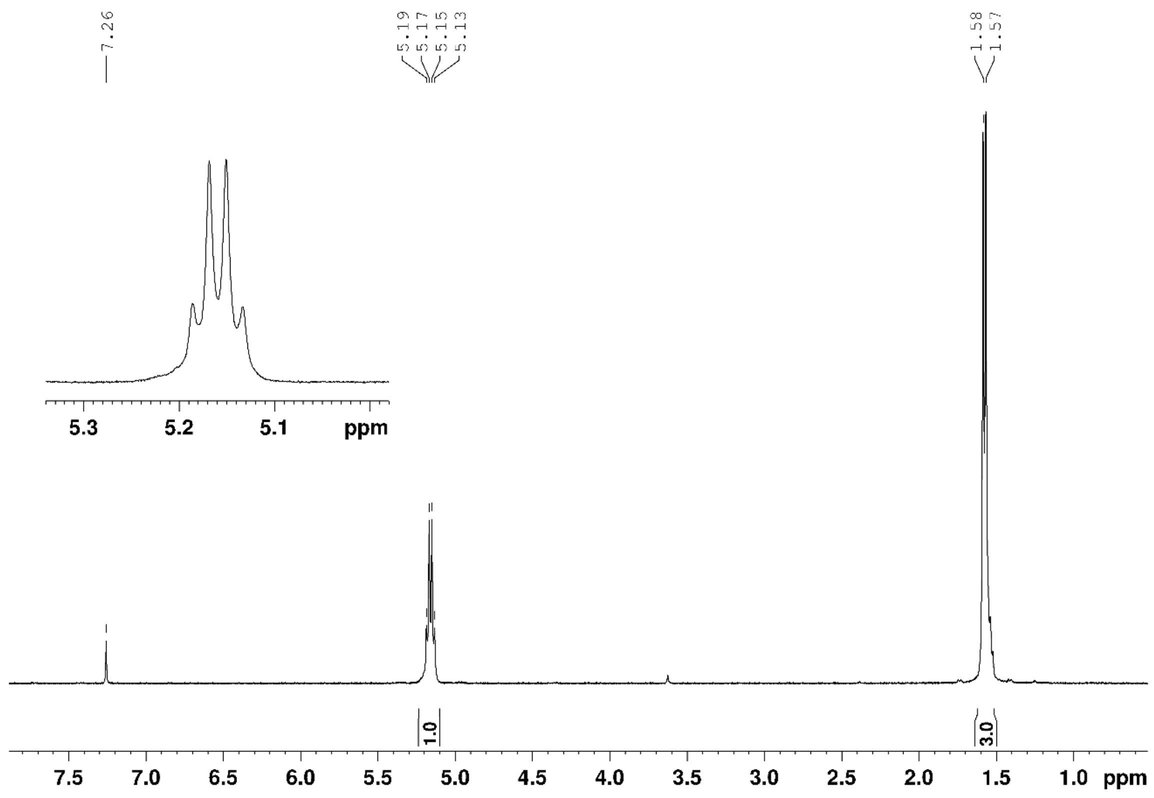

Figure S6. ^{1}H NMR (298K, $CDCl_3$): Spectrum for washed polymer of enter 20 (**2**:LLA:BnOH ratio 1:200:0).

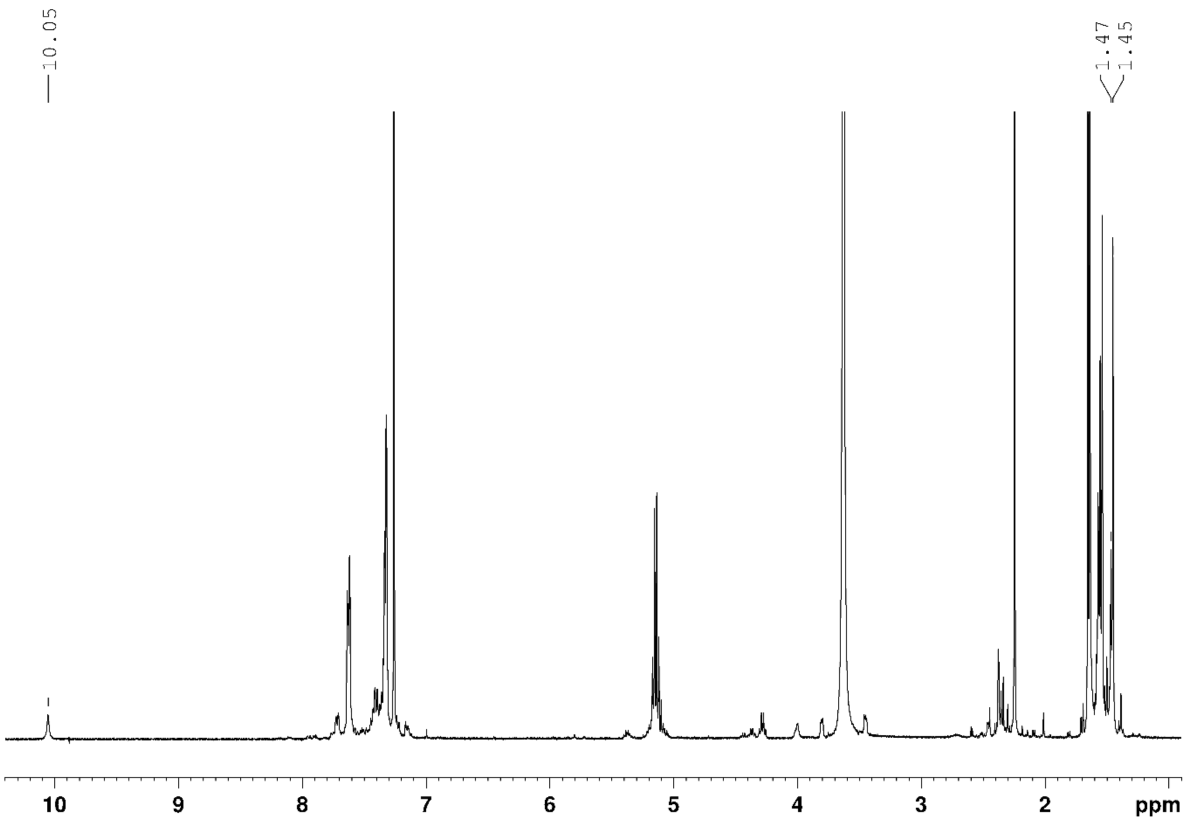

Figure S7. ^{1}H NMR (298K, $CDCl_3$): Study of **2**:LLA:BnOH ratio 1:1:0.

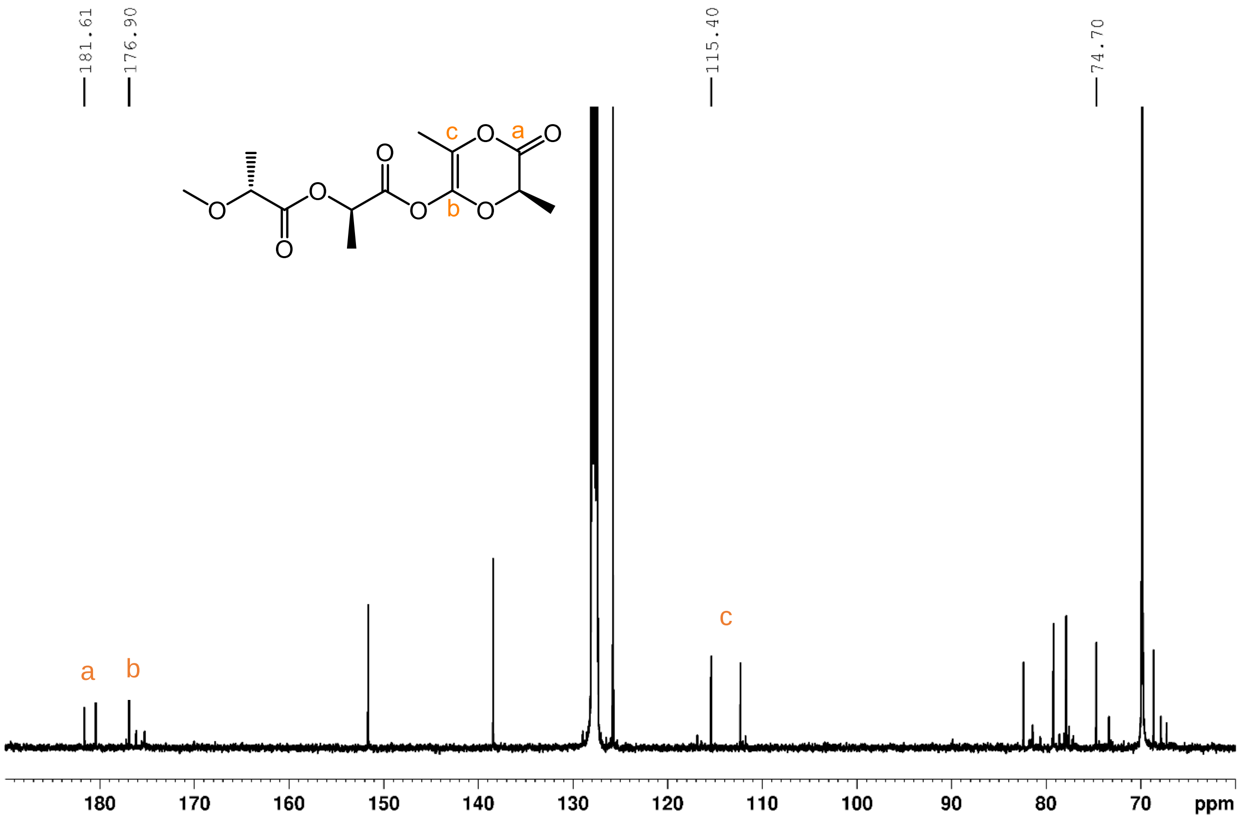

Figure S8. ^{13}C {^{1}H} NMR (298K, C_6D_6): Study of **2**:LLA:BnOH ratio 1:1:0.

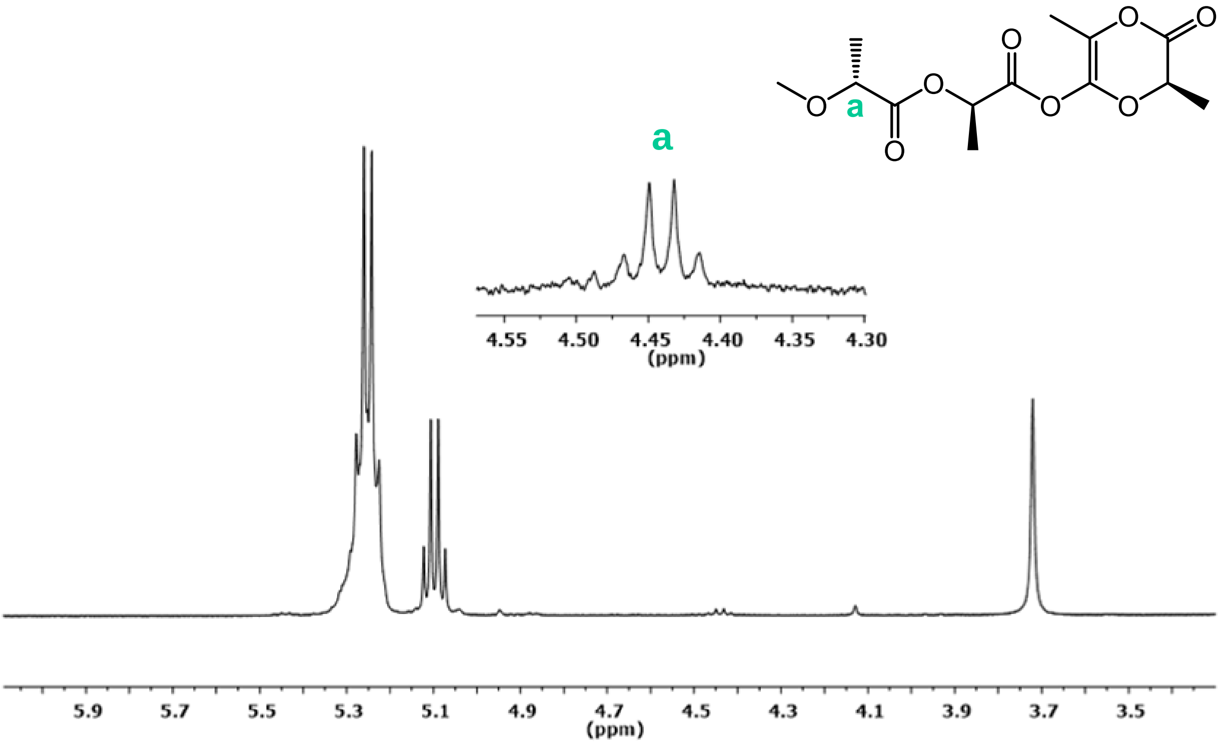

Figure S9. ^{1}H NMR (298 K, C_6D_6): Study of (1S,4R)-[K(18-crown-6)(L)]:LLA:BnOH ratio 1:100:0 at -70 °C, before reaching full conversion.

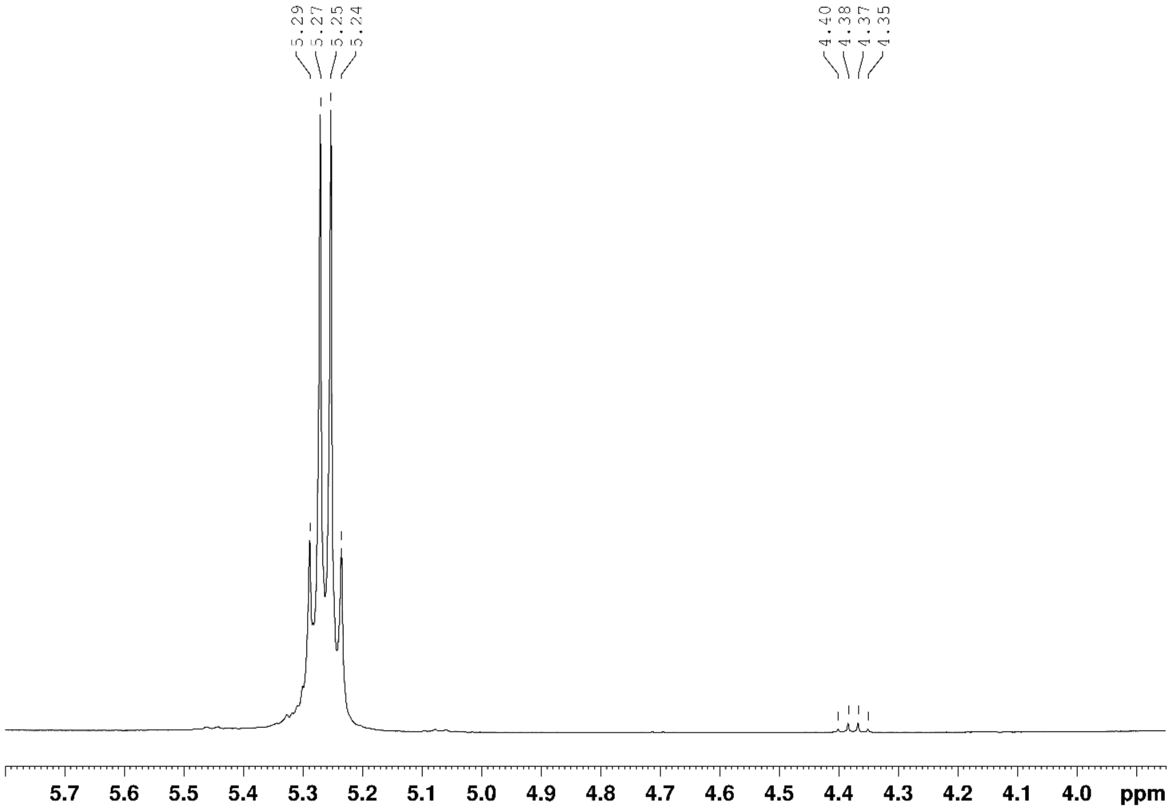

Figure S10. ^{1}H NMR (298K, $CDCl_3$): Spectrum ampliation for washed polymer of enter 20 (**2**:LLA:BnOH ratio 1:200:0).

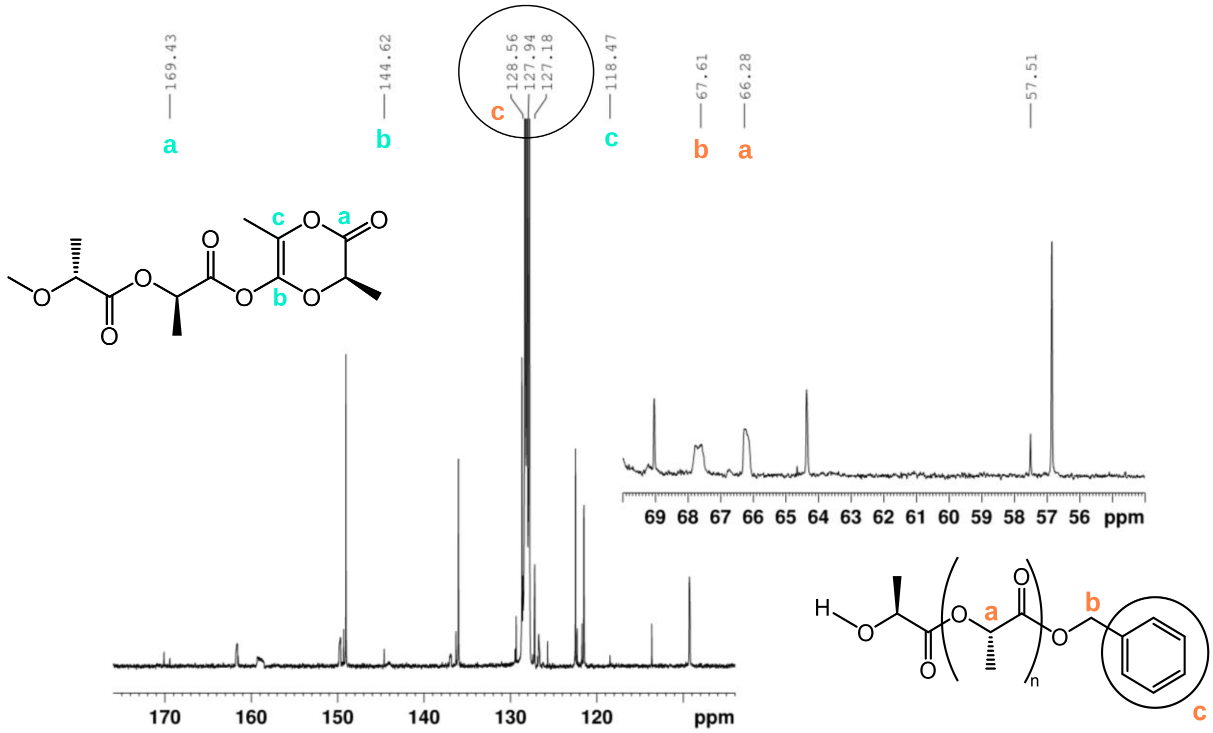

Figure S11. ^{13}C NMR spectrum for the (1*S*,4*R*)-[K(18-crown-6)(L)]:LLA:BnOH ratio 1:1:1 in C_6D_6. The signals corresponding to the aromatic carbons from the benzyl termination are partially obscured by the solvent ones.

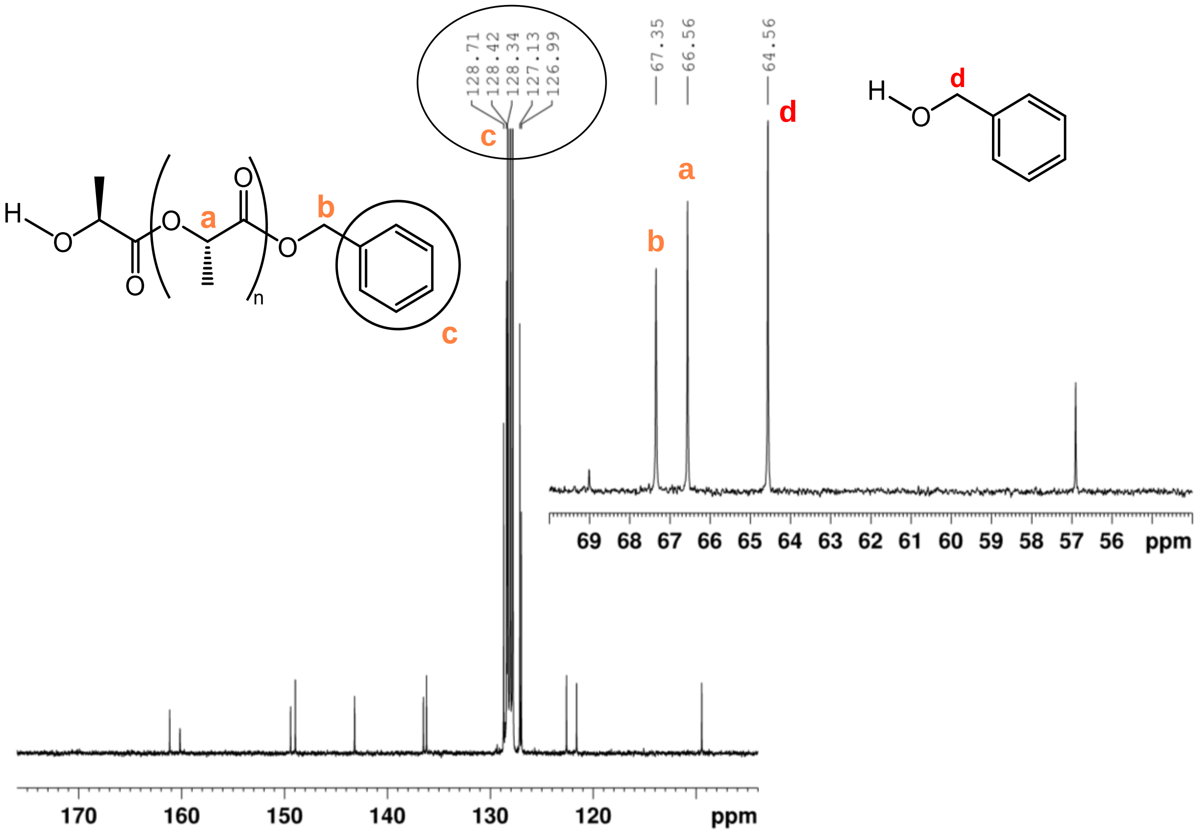

Figure S12. ^{13}C NMR spectrum for the (1*S*,4*R*)-[K(18-crown-6)(L)]:LLA:BnOH ratio 1:1:5 in C_6D_6. The signals corresponding to the aromatic carbons from the benzyl termination are partially obscured by the solvent ones.

2. Diffusion-Ordered NMR Spectroscopy (DOSY)

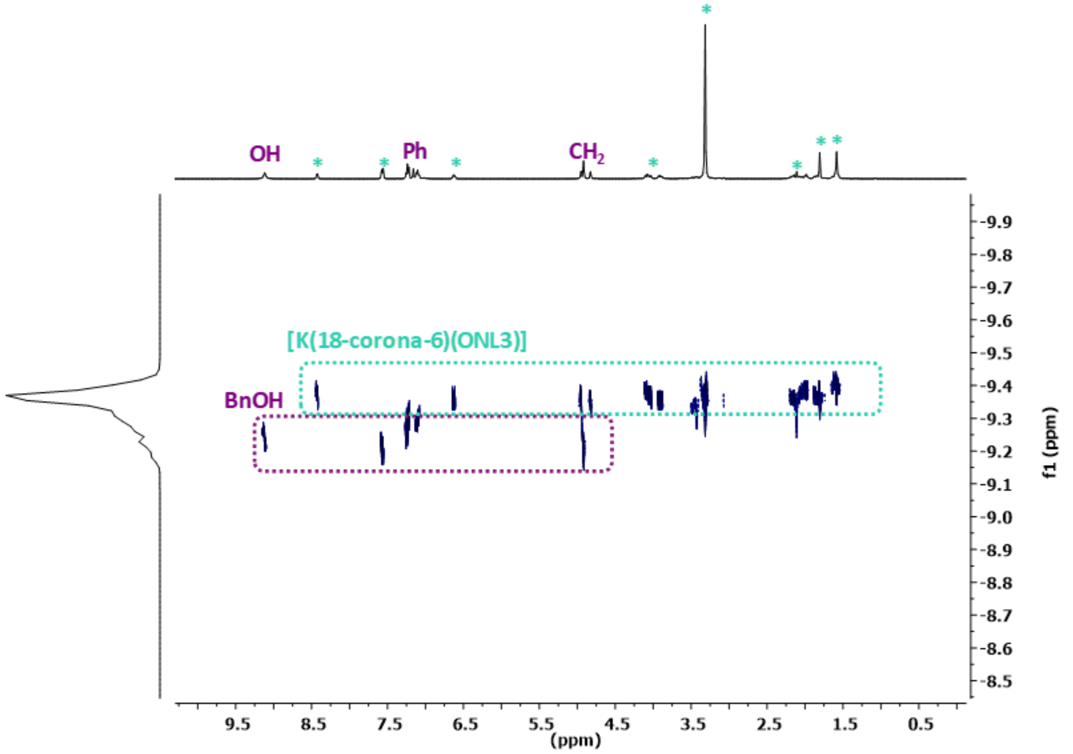

Figure S13. 2D ^{1}H DOSY NMR experiment for the (1S,4R)-[K(18-crown-6)(L)]/BnOH mixture.

3. Single-Crystal X-Ray Structure Determination

Table S1 Crystal data and structure refinement for 2 and 2·H₂O

	**2**	**2.H₂O**
Empirical formula	$C_{20}H_{30}KNO_7$	$C_{20}H_{34}KNO_8$
Formula weight	435.55	455.58
Temperature/K	200.0	200.0
Crystal system	orthorhombic	monoclinic
Space group	$Pca2_1$	$P2_1/n$
a/Å	22.9351(12)	13.8657(14)
b/Å	8.3809(4)	8.7497(8)
c/Å	23.6975(15)	19.4651(19)
α/°	90	90
β/°	90	93.772(4)
γ/°	90	90
Volume/$Å^3$	4555.1(4)	2356.4(4)
Z	8	4
ρ_{calc} g/cm^3	1.270	1.284
μ/mm^{-1}	0.271	0.268
F(000)	1856.0	976.0
Crystal size/mm^3	0.34 × 0.32 × 0.22	0.37 × 0.25 × 0.23
Radiation	MoKα (λ = 0.71073)	MoKα (λ = 0.71073)
2Θ range for data collection/°	3.946 to 57.666	5.508 to 56.504
Index ranges	$-30 \le h \le 29$, $-9 \le k \le 11$, $-32 \le l \le 32$	$-17 \le h \le 17$, $-11 \le k \le 9$, $-23 \le l \le 25$
Reflections collected	36581	15956
Independent reflections	10517 [R_{int} = 0.1034]	5319 [R_{int} = 0.1079]
Data/restraints/parameters	10517/1/526	5319/0/272
Goodness-of-fit on F^2	1.056	1.025
Final R indexes [I>=2σ (I)]	R_1 = 0.0619, wR_2 = 0.1381	R_1 = 0.0584, wR_2 = 0.1183
Final R indexes [all data]	R_1 = 0.1412, wR_2 = 0.1794	R_1 = 0.1282, wR_2 = 0.1415
Largest diff. peak/hole / e $Å^{-3}$	0.71/-0.67	0.29/-0.41
Flack parameter	0.01(9)	
CCDC	2181870	2181869

Table S2: Bond Lengths (Å) and Angles (°) for 2.

K1	O13	3.060(5)	O14	C43	1.416(8)
K1	O11	2.978(5)	O14	C32	1.406(8)
K1	O10	2.834(5)	O7	C30	1.421(9)
K1	O12	2.849(4)	O7	C29	1.436(9)
K1	O1	2.637(5)	O2	N2	1.332(7)
K1	O9	2.970(5)	N2	C1	1.317(8)
K1	O14	2.894(5)	O3	C26	1.433(8)
K1	N1	2.817(6)	O3	C25	1.423(8)
K1	C38	3.533(8)	C23	C22	1.499(11)
K2	O6	2.936(5)	C20	C21	1.499(10)
K2	O8	2.932(4)	N1	C11	1.308(9)
K2	O4	3.093(5)	C3	C8	1.390(10)
K2	O5	2.852(4)	C3	C4	1.421(10)
K2	O7	2.818(5)	C3	C1	1.481(10)
K2	O2	2.609(5)	C27	C26	1.485(10)
K2	N2	2.799(6)	C41	C40	1.484(10)
K2	O3	2.887(5)	C8	C7	1.372(11)
K2	C20	3.517(7)	C30	C31	1.485(11)
K2	C31	3.538(7)	C35	C34	1.465(12)
O6	C20	1.434(8)	C4	C5	1.369(11)
O6	C31	1.415(8)	C1	C2	1.477(9)
O13	C41	1.410(8)	C13	C14	1.392(10)
O13	C42	1.421(8)	C13	C18	1.398(10)
O8	C27	1.432(8)	C13	C11	1.460(10)
O8	C28	1.425(8)	C39	C38	1.480(10)
O4	C23	1.427(8)	C14	C15	1.392(11)
O4	C24	1.414(9)	C29	C28	1.504(11)
O11	C38	1.416(9)	C15	C16	1.362(13)
O11	C37	1.446(9)	C36	C37	1.497(12)
O10	C35	1.402(9)	C18	C17	1.390(11)
O10	C36	1.400(10)	C42	C43	1.479(10)
O12	C39	1.423(8)	C7	C6	1.358(12)
O12	C40	1.405(8)	C24	C25	1.496(10)
O5	C22	1.403(8)	C11	C12	1.515(10)
O5	C21	1.431(8)	C16	C17	1.352(13)
O1	N1	1.328(7)	C6	C5	1.416(13)
O9	C34	1.409(9)	C33	C32	1.483(11)

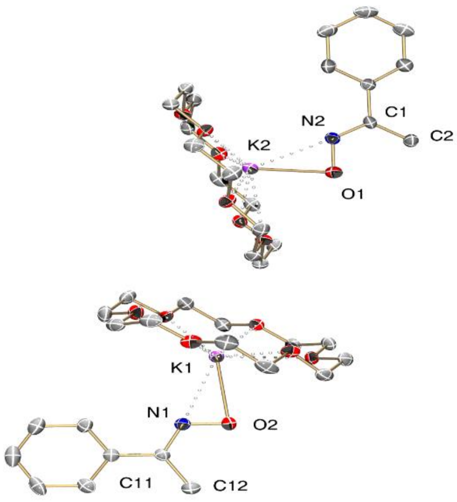

O9	C33	1.429(10)					
O13	K1	C38	97.03(15)	C28	O8	C27	111.1(5)
O11	K1	O13	106.67(13)	C23	O4	K2	107.4(4)
O11	K1	C38	23.17(16)	C24	O4	K2	107.6(4)
O10	K1	O13	142.72(14)	C24	O4	C23	112.0(6)
O10	K1	O11	58.99(15)	C38	O11	K1	101.0(4)
O10	K1	O12	117.21(16)	C38	O11	C37	110.7(6)
O10	K1	O9	56.37(15)	C37	O11	K1	102.0(4)
O10	K1	O14	112.34(15)	C35	O10	K1	122.4(4)
O10	K1	C38	79.52(18)	C36	O10	K1	119.7(4)
O12	K1	O13	56.32(12)	C36	O10	C35	113.6(6)
O12	K1	O11	58.61(14)	C39	O12	K1	118.3(4)
O12	K1	O9	145.86(15)	C40	O12	K1	123.4(4)
O12	K1	O14	112.12(13)	C40	O12	C39	112.7(5)
O12	K1	C38	41.78(16)	C22	O5	K2	124.7(4)
O1	K1	O13	119.54(15)	C22	O5	C21	111.4(5)
O1	K1	O11	81.37(16)	C21	O5	K2	117.3(4)
O1	K1	O10	93.61(16)	N1	O1	K1	83.6(3)
O1	K1	O12	83.64(14)	C34	O9	K1	108.3(4)
O1	K1	O9	128.07(14)	C34	O9	C33	112.9(6)
O1	K1	O14	135.73(17)	C33	O9	K1	107.5(4)
O1	K1	N1	27.95(15)	C43	O14	K1	122.8(4)
O1	K1	C38	67.30(17)	C32	O14	K1	120.7(4)
O9	K1	O13	106.36(13)	C32	O14	C43	111.2(5)
O9	K1	O11	108.60(15)	C30	O7	K2	119.9(4)
O9	K1	C38	131.76(18)	C30	O7	C29	113.3(6)
O14	K1	O13	56.01(12)	C29	O7	K2	121.9(4)
O14	K1	O11	142.59(14)	N2	O2	K2	83.8(3)
O14	K1	O9	56.61(14)	O2	N2	K2	67.9(3)
O14	K1	C38	149.48(16)	C1	N2	K2	175.7(5)
N1	K1	O13	131.22(15)	C1	N2	O2	116.1(6)
N1	K1	O11	100.37(15)	C26	O3	K2	117.3(4)
N1	K1	O10	86.05(16)	C25	O3	K2	124.0(4)
N1	K1	O12	111.00(15)	C25	O3	C26	111.5(5)
N1	K1	O9	102.22(15)	O4	C23	C22	107.5(6)
N1	K1	O14	115.85(16)	O6	C20	K2	54.8(3)
N1	K1	C38	92.26(17)	O6	C20	C21	109.2(5)

O6	K2	O4	108.54(13)	C21	C20	K2	86.2(4)
O6	K2	C20	23.53(15)	O1	N1	K1	68.5(3)
O6	K2	C31	22.91(14)	C11	N1	K1	173.6(5)
O8	K2	O6	108.74(15)	C11	N1	O1	117.5(5)
O8	K2	O4	107.79(12)	C8	C3	C4	116.6(7)
O8	K2	C20	132.28(16)	C8	C3	C1	122.2(6)
O8	K2	C31	98.18(16)	C4	C3	C1	121.3(6)
O4	K2	C20	96.91(15)	O8	C27	C26	109.1(6)
O4	K2	C31	131.42(15)	O13	C41	C40	109.6(6)
O5	K2	O6	59.73(14)	C7	C8	C3	122.1(7)
O5	K2	O8	145.92(14)	O5	C22	C23	108.6(5)
O5	K2	O4	55.54(12)	O7	C30	C31	108.5(6)
O5	K2	O3	110.69(13)	O6	C31	K2	53.9(3)
O5	K2	C20	42.04(16)	O6	C31	C30	110.5(6)
O5	K2	C31	80.04(16)	C30	C31	K2	85.8(4)
O7	K2	O6	58.76(14)	O10	C35	C34	109.3(6)
O7	K2	O8	57.42(14)	C5	C4	C3	120.7(8)
O7	K2	O4	147.18(14)	N2	C1	C3	117.0(6)
O7	K2	O5	118.22(15)	N2	C1	C2	122.3(6)
O7	K2	O3	116.04(14)	C2	C1	C3	120.6(6)
O7	K2	C20	79.71(16)	O5	C21	C20	107.8(5)
O7	K2	C31	41.65(16)	C14	C13	C18	116.4(7)
O2	K2	O6	79.56(15)	C14	C13	C11	121.9(6)
O2	K2	O8	127.41(14)	C18	C13	C11	121.7(7)
O2	K2	O4	118.60(15)	O9	C34	C35	108.3(6)
O2	K2	O5	84.17(14)	O12	C39	C38	109.0(6)
O2	K2	O7	90.24(16)	C15	C14	C13	121.8(8)
O2	K2	N2	28.24(15)	O7	C29	C28	107.8(6)
O2	K2	O3	134.44(17)	C16	C15	C14	120.1(8)
O2	K2	C20	66.52(16)	O10	C36	C37	109.9(7)
O2	K2	C31	69.28(17)	O11	C38	K1	55.8(3)
N2	K2	O6	100.95(15)	O11	C38	C39	109.5(6)
N2	K2	O8	102.71(15)	C39	C38	K1	86.2(4)
N2	K2	O4	127.04(15)	C17	C18	C13	120.7(8)
N2	K2	O5	110.84(15)	O8	C28	C29	107.1(6)
N2	K2	O7	85.77(15)	O13	C42	C43	109.5(6)
N2	K2	O3	112.91(16)	O3	C26	C27	109.3(5)

N2	K2	C20	93.14(17)	C6	C7	C8	121.7(8)
N2	K2	C31	83.80(17)	O4	C24	C25	108.1(6)
O3	K2	O6	145.56(14)	N1	C11	C13	117.5(6)
O3	K2	O8	58.85(13)	N1	C11	C12	120.5(6)
O3	K2	O4	55.33(12)	C13	C11	C12	121.9(6)
O3	K2	C20	149.65(16)	O14	C43	C42	110.0(5)
O3	K2	C31	153.25(17)	C17	C16	C15	119.5(8)
C20	K2	C31	38.74(17)	O11	C37	C36	109.6(6)
C20	O6	K2	101.6(4)	O12	C40	C41	108.8(6)
C31	O6	K2	103.2(4)	C7	C6	C5	117.9(8)
C31	O6	C20	110.5(5)	O3	C25	C24	108.5(6)
C41	O13	K1	105.9(4)	C16	C17	C18	121.4(9)
C41	O13	C42	112.7(5)	O9	C33	C32	107.8(6)
C42	O13	K1	107.9(4)	C4	C5	C6	121.0(8)
C27	O8	K2	107.0(4)	O14	C32	C33	109.0(6)
C28	O8	K2	108.6(4)				

Table S3: Bond Lengths (Å) and Angles (°) for 2·H_2O.

K1	N1	2.947(2)	O14	C17	1.405(3)
K1	O1	2.784(2)	O15	C18	1.423(3)
K1	O2	2.8183(19)	O15	C19	1.414(4)
K1	O11	3.0010(19)	O16	C20	1.415(3)
K1	O12	2.7600(19)	O16	C21	1.414(3)
K1	O13	3.033(2)	C1	C2	1.470(4)
K1	O14	2.9323(19)	C1	C10	1.499(4)
K1	O15	3.0249(19)	C2	C3	1.405(4)
K1	O16	2.8892(19)	C2	C7	1.393(4)
K1	C11	3.540(3)	C3	C4	1.380(4)
K1	C14	3.522(3)	C4	C5	1.378(4)
N1	O1	1.339(3)	C5	C6	1.379(4)
N1	C1	1.304(3)	C6	C7	1.381(4)
O11	C11	1.429(3)	C11	C12	1.491(4)
O11	C22	1.436(3)	C13	C14	1.482(4)
O12	C12	1.417(3)	C15	C16	1.481(4)
O12	C13	1.418(3)	C17	C18	1.485(5)
O13	C14	1.431(3)	C19	C20	1.495(4)
O13	C15	1.418(3)	C21	C22	1.490(4)

O14	C16		1.427(3)				
N1	K1	O11	98.92(6)	O16	K1	C11	78.38(6)
N1	K1	O13	135.76(6)	O16	K1	C14	153.24(7)
N1	K1	O15	96.59(6)	C14	K1	C11	82.91(7)
N1	K1	C11	91.46(6)	O1	N1	K1	69.78(12)
N1	K1	C14	119.54(6)	C1	N1	K1	126.92(16)
O1	K1	N1	26.84(5)	C1	N1	O1	117.6(2)
O1	K1	O2	74.37(5)	N1	O1	K1	83.38(13)
O1	K1	O11	73.82(5)	C11	O11	K1	99.96(15)
O1	K1	O13	136.74(5)	C11	O11	C22	110.2(2)
O1	K1	O14	141.22(6)	C22	O11	K1	105.29(15)
O1	K1	O15	113.00(6)	C12	O12	K1	122.87(15)
O1	K1	O16	75.09(5)	C12	O12	C13	114.3(2)
O1	K1	C11	64.63(6)	C13	O12	K1	122.15(16)
O1	K1	C14	113.61(6)	C14	O13	K1	97.66(15)
O2	K1	N1	59.95(6)	C15	O13	K1	104.52(15)
O2	K1	O11	135.28(5)	C15	O13	C14	111.0(2)
O2	K1	O13	76.42(5)	C16	O14	K1	118.32(16)
O2	K1	O14	77.41(5)	C17	O14	K1	120.47(17)
O2	K1	O15	113.17(5)	C17	O14	C16	112.2(2)
O2	K1	O16	138.08(6)	C18	O15	K1	109.14(15)
O2	K1	C11	112.60(6)	C19	O15	K1	106.56(16)
O2	K1	C14	67.27(6)	C19	O15	C18	113.9(2)
O11	K1	O13	107.77(5)	C20	O16	K1	122.50(16)
O11	K1	O15	107.80(5)	C21	O16	K1	118.05(15)
O11	K1	C11	23.43(5)	C21	O16	C20	112.9(2)
O11	K1	C14	98.39(6)	N1	C1	C2	115.8(2)
O12	K1	N1	114.74(6)	N1	C1	C10	122.2(2)
O12	K1	O1	92.73(6)	C2	C1	C10	121.8(2)
O12	K1	O2	93.86(6)	C3	C2	C1	120.9(2)
O12	K1	O11	57.44(5)	C7	C2	C1	121.8(3)
O12	K1	O13	58.20(5)	C7	C2	C3	117.2(3)
O12	K1	O14	115.34(6)	C4	C3	C2	121.1(3)
O12	K1	O15	146.36(6)	C5	C4	C3	120.4(3)
O12	K1	O16	115.63(6)	C4	C5	C6	119.6(3)
O12	K1	C11	41.70(6)	C5	C6	C7	120.3(3)
O12	K1	C14	41.67(6)	C6	C7	C2	121.4(3)

O13	K1	C11	99.22(6)	O11	C11	K1	56.61(12)
O13	K1	C14	23.75(6)	O11	C11	C12	108.6(2)
O14	K1	N1	114.58(6)	C12	C11	K1	85.21(15)
O14	K1	O11	143.46(6)	O12	C12	C11	108.3(2)
O14	K1	O13	57.47(5)	O12	C13	C14	107.9(2)
O14	K1	O15	56.25(5)	O13	C14	K1	58.60(12)
O14	K1	C11	153.00(7)	O13	C14	C13	109.8(2)
O14	K1	C14	78.14(6)	C13	C14	K1	85.56(15)
O15	K1	O13	107.53(6)	O13	C15	C16	109.5(2)
O15	K1	C11	130.99(6)	O14	C16	C15	109.1(2)
O15	K1	C14	131.14(6)	O14	C17	C18	108.5(2)
O16	K1	N1	80.16(6)	O15	C18	C17	108.6(3)
O16	K1	O11	58.46(5)	O15	C19	C20	108.1(2)
O16	K1	O13	144.00(6)	O16	C20	C19	108.5(2)
O16	K1	O14	111.66(5)	O16	C21	C22	109.4(2)
O16	K1	O15	55.89(5)	O11	C22	C21	108.8(2)

4. Density Functional Theory (DFT) calculations

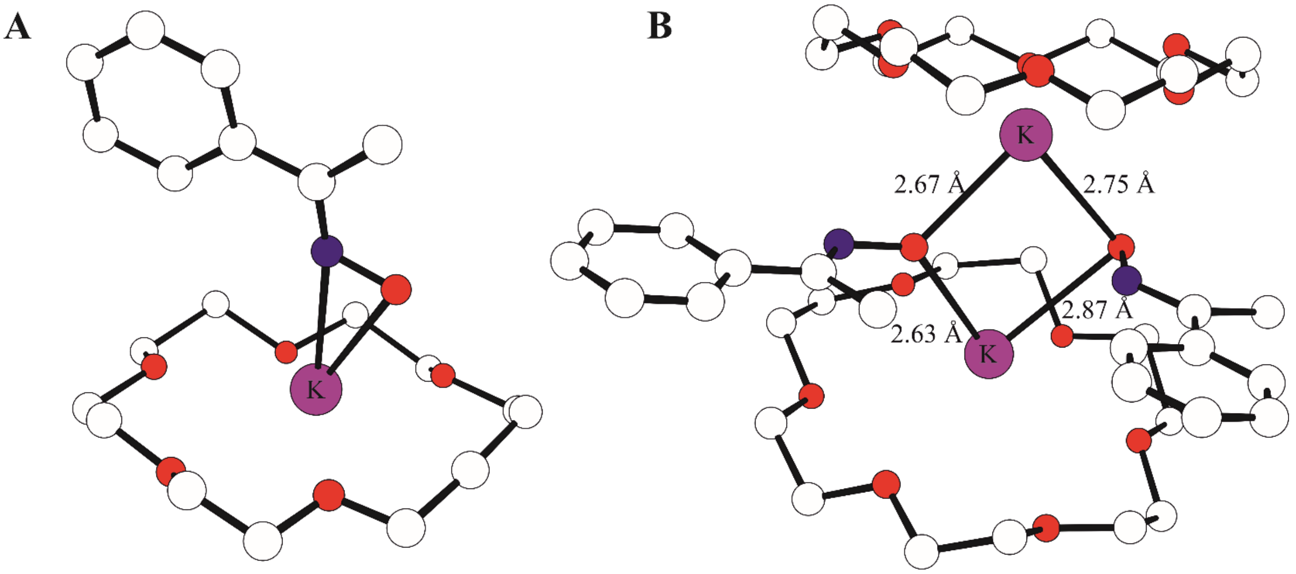

Figure S14. DFT geometries of system **1** optimized in gas-phase for mononuclear (**A**) and dinuclear (**B**) species. The calculated Gibbs energies are corrected by BSSE (ΔG_{corr} = 1.8 kcal/mol) showing a preference for mononuclear specie (**A**). Hydrogen atoms omitted for clarity.

5. Polymer characterization

5.1. Gel Permeation Cromatography (GPC)

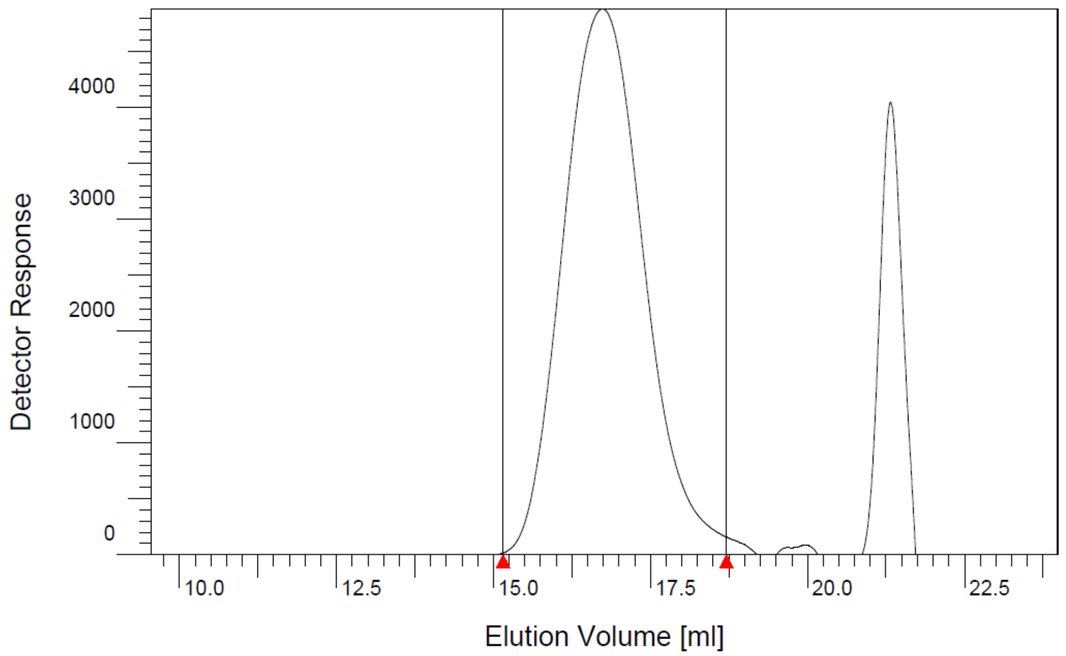

Figure S15. Chromatogram example for polymer of enter 6 (**2**:LLA:BnOH ratio 1:200:5).

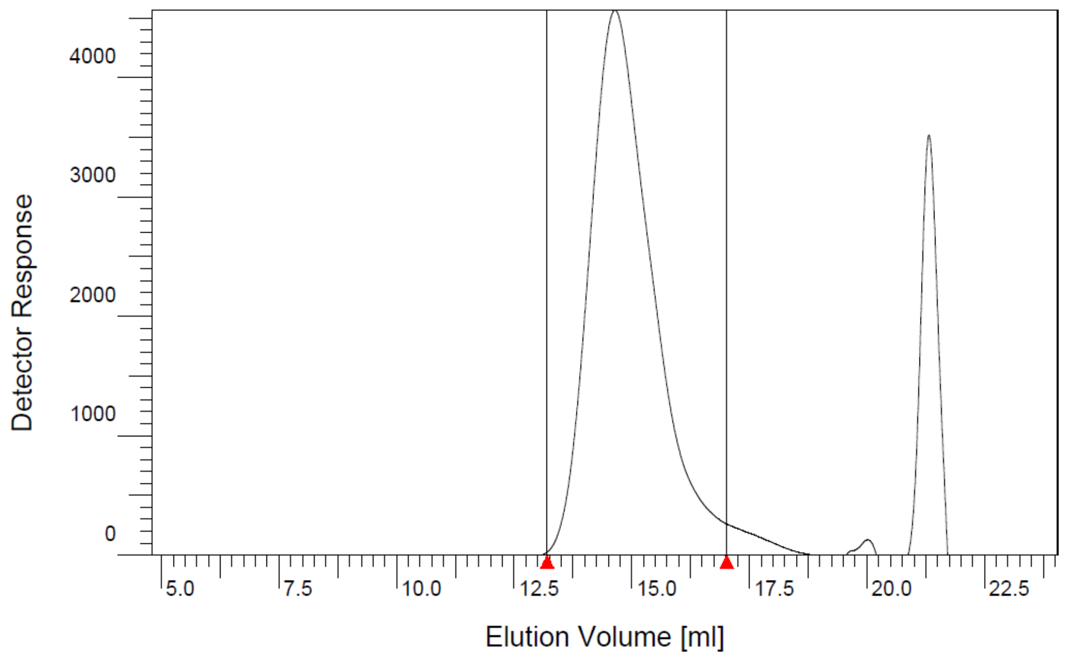

Figure S16. Chromatogram example for polymer of enter 20 (**2**:LLA:BnOH ratio 1:200:0).

4.2. Mass Spectroscopy (MS)

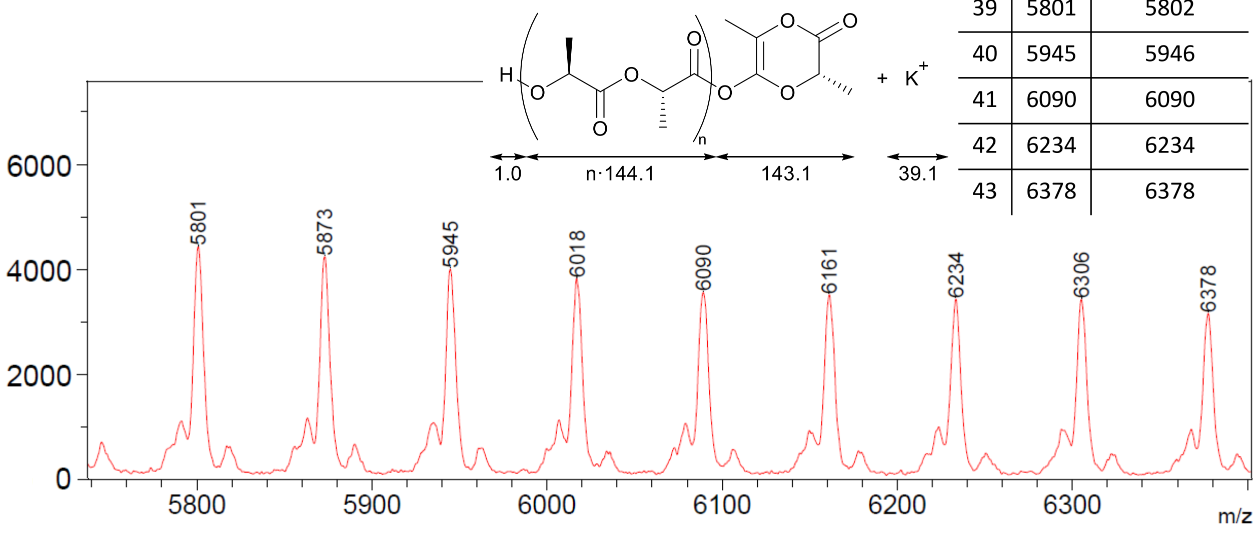

n	m/z	Theo. Values
39	5801	5802
40	5945	5946
41	6090	6090
42	6234	6234
43	6378	6378

Figure S17. MALDI-TOF spectrum for polymer of enter 20 (**2**:LLA:BnOH ratio 1:200:0).

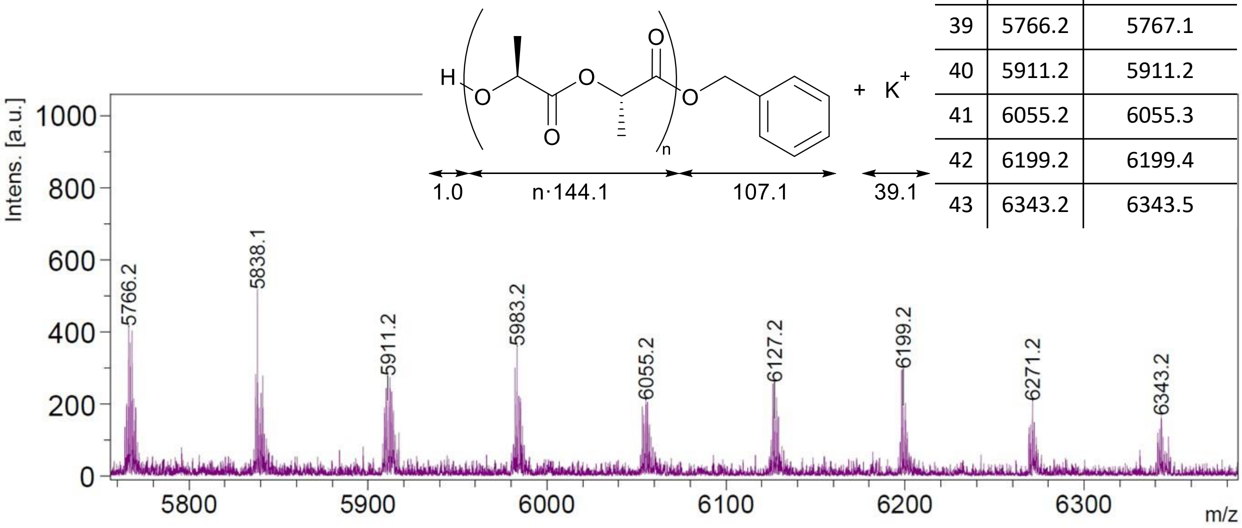

n	m/z	Theo. Values
39	5766.2	5767.1
40	5911.2	5911.2
41	6055.2	6055.3
42	6199.2	6199.4
43	6343.2	6343.5

Figure S18. MALDI-TOF spectrum for polymer of enter 6 (**2**:LLA:BnOH ratio 1:200:5).

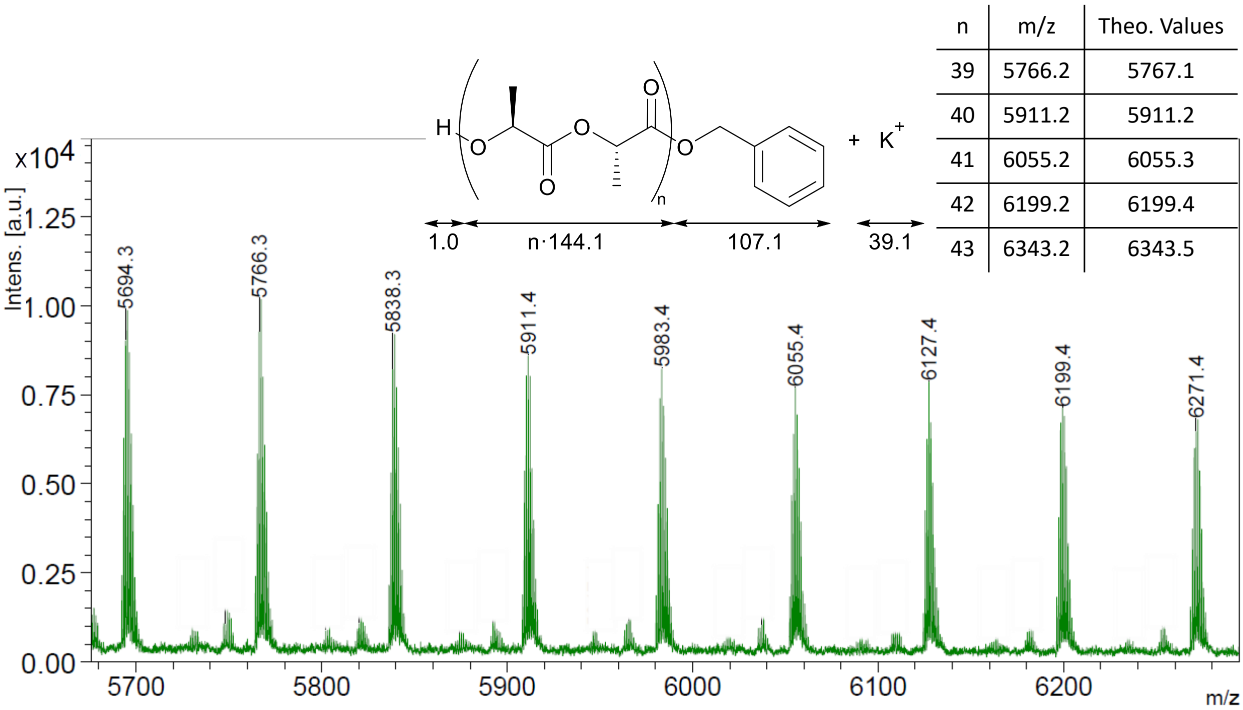

n	m/z	Theo. Values
39	5766.2	5767.1
40	5911.2	5911.2
41	6055.2	6055.3
42	6199.2	6199.4
43	6343.2	6343.5

Figure S19. MALDI-TOF spectrum for polymer of enter 12 (**2**:LLA:BnOH ratio 1:200:2).
